# Sex differences in ectopic lipid deposits and cardiac function across a wide range of glycemic control: a secondary analysis

**DOI:** 10.1002/oby.24153

**Published:** 2024-11-18

**Authors:** Jürgen Harreiter, Ivica Just, Michael Weber, Radka Klepochová, Magdalena Bastian, Yvonne Winhofer, Peter Wolf, Thomas Scherer, Michael Leutner, Lana Kosi‐Trebotic, Carola Deischinger, Marek Chmelík, Michael R. Krebs, Siegfried Trattnig, Martin Krššák, Alexandra Kautzky‐Willer

**Affiliations:** ^1^ Division of Endocrinology and Metabolism, Department of Internal Medicine III Medical University of Vienna Vienna Austria; ^2^ Department of Medicine Landesklinikum Scheibbs Scheibbs Austria; ^3^ High Field MR Center, Department of Biomedical Imaging and Image‐guided Therapy Medical University of Vienna Vienna Austria; ^4^ Department of Biomedical Imaging and Image‐guided Therapy Medical University of Vienna Vienna Austria; ^5^ Department of Technical Disciplines in Health Care at Faculty of Health Care University of Prešov Prešov Slovakia

## Abstract

**Objective:**

The objective of this study was to identify sex differences in ntrahepatocellular (HCL) and intramyocardial lipids (MYCL) and cardiac function in participants with different grades of glucometabolic impairment and different BMI strata.

**Methods:**

Data from 503 individuals from 17 clinical experimental studies were analyzed. HCL and MYCL were assessed with 3T and 7T scanners by magnetic resonance spectroscopy. Cardiac function was measured with a 3T scanner using electrocardiogram‐gated TrueFISP sequences. Participants were classified as having normoglycemia, prediabetes, or type 2 diabetes. Three‐way ANCOVA with post hoc simple effects analyses was used for statistical assessment.

**Results:**

Consistent increases of HCL with BMI and deterioration of glucose metabolism, especially in female individuals, were detected. MYCL increased with BMI and glucose impairment in female individuals, but not in male individuals. Sex differences were found in cardiac function loss, with significant effects found among male individuals with worsening glucose metabolism. Myocardial mass and volume of the ventricle were higher in male individuals in all groups. This sex difference narrowed with increasing BMI and with progressing dysglycemia.

**Conclusions:**

Sex differences in HCL and MYCL may be associated with a higher cardiovascular disease risk observed in female individuals progressing to diabetes. Further studies are needed to elucidate possible sex differences with advancing glucometabolic impairment and obesity and their potential impact on cardiovascular outcomes.

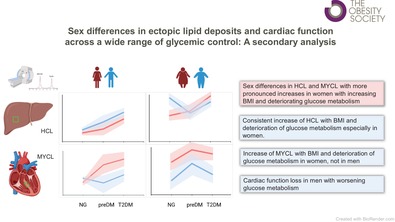

## Graphical abstract

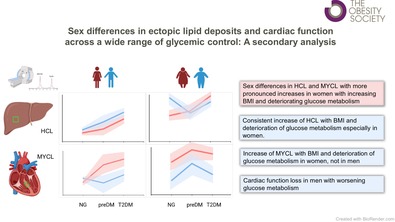

**Study Importance**

**What is already known?**

- Sex differences in diabetes, obesity, metabolic disorders, and cardiovascular disease have been well described.
- Differences between female and male individuals in intrahepatocellular (HCL) and intramyocardial lipids (MYCL) with increasing impairment of glucose metabolism and different BMI strata are controversial and still not well investigated.

**What does this study add?**

- A consistent increase of HCL with BMI and deterioration of glucose metabolism was observed, especially in female individuals.
- An increase of MYCL with BMI and deterioration of glucose metabolism was observed in female individuals, but not in male individuals.
- Cardiac function loss in male individuals was observed with worsening glucose metabolism.

**How might these results change the direction of research or the focus of clinical practice?**

- Sex differences in HCL and MYCL may be associated with a higher cardiovascular disease risk observed in female individuals progressing to diabetes.

## INTRODUCTION

Differences between male and female individuals have been well described in the presence of diabetes, obesity (OB), metabolic disorders, and associated cardiovascular disease. Therefore, these essential risk factors might also contribute to sex differences in intramyocardial (MYCL) or intrahepatocellular lipid (HCL) accumulation, cardiac function, and subsequent cardiovascular outcome [1]. Various risk factors are relevant for the accumulation of ectopic lipids in the liver, heart, or other organs; among those are the main contributors to fatty liver disease, OB, and metabolic disorders such as diabetes, which recently led to the new terminology, i.e., metabolic dysfunction‐associated steatotic liver disease (MASLD). Independent of other risk factors such as overweight and OB, hyperglycemia, hypertension, hyperlipidemia, or sedentary behavior, MASLD per se was associated with around a 30% to 50% higher risk for all‐cause, cardiac, and cancer mortality [2].

During the reproductive age, more male individuals are affected by MASLD, and the severity of the disease is higher in male individuals [3]. This changes after menopause due to the loss of potential beneficial effects of female sex hormones and/or the increased accumulation of risk factors in female individuals attributable to hormonal and metabolic changes. A higher risk for MASLD was observed in female individuals with elevated glucose parameters, presumably associated with the worsening of concomitant metabolic risk factors [1]. However, recent studies have been controversial and either confirm a higher risk of MASLD in female individuals with progressing dysglycemia than male individuals or show no differences in HCL between sexes [4, 5].

Increased MYCL was reported in individuals with insulin resistance, OB, and diabetes, as well as in those with increasing age, and is a relevant determinant of cardiac function [6]. Elevation of MYCL was associated with a higher risk of cardiac remodeling and left ventricular dysfunction. In female individuals, risk factors such as OB and insulin resistance, which are associated with diastolic ventricular dysfunction and type 2 diabetes mellitus (T2DM), were found to have more severe effects on progression to heart failure [1]. Whether there are sex differences in MYCL is controversial. In healthy individuals with normal weight, no sex differences in MYCL were reported [7]. Other studies have reported that female individuals present with lower myo‐ and pericardial lipids than male individuals, which might revert with progression from normoglycemia (NG) to dysglycemia [8]. Further research is needed to better understand these differences and their implications for individualized disease management.

In this secondary analysis, we aimed to identify differences between male and female individuals in HCL and MYCL and ventricular function parameters assessed by magnetic resonance (MR) spectroscopy/imaging (MRI) across different stages of glucose metabolism impairment and different body mass index (BMI) strata.

## METHODS

### Participants

This was a cross‐sectional analysis of selected cohorts of individuals from 17 clinical experimental studies performed at the Medical University of Vienna, Austria, at the Division of Endocrinology and Metabolism and the High Field MR Center from 2008 to 2022. Individual data from all except four studies have been published [9, 10, 11, 12, 13, 14, 15, 16, 17, 18, 19, 20, 21]. All studies comply with the Declaration of Helsinki and were approved by the local institutional ethics committee (ethics committees of Medical University Vienna and Medical University Graz). Corresponding ethics numbers can be found in online Supporting Information. Written informed consent was obtained from all participants prior to study‐related activities.

**FIGURE 1:**
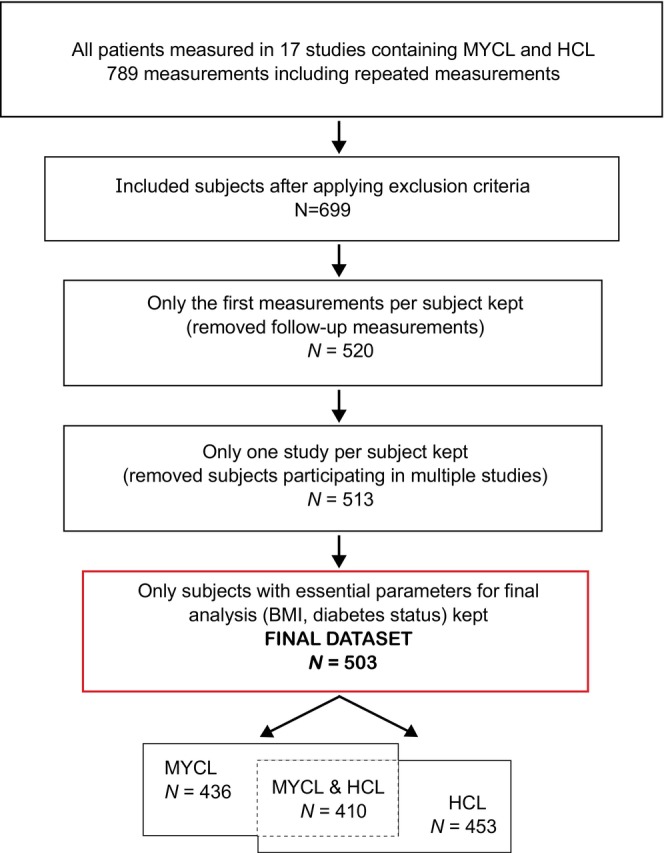
Process of selection of the cohort for cross‐sectional analysis. HCL, intrahepatocellular lipid; MYCL, intramyocardial lipids.

The cohort for this analysis was selected from the studies with MR acquisition of MYCL or HCL. Depending on the nature and inclusion criteria of the individual study, either all participants or only healthy volunteers were included. Individuals with metabolic dysfunction‐associated fatty liver disease, metabolic syndrome, prediabetes (preDM), T2DM, and polycystic ovary syndrome were included in the analysis. Patients excluded from our cross‐sectional analysis had alterations in calcium metabolism, type 1 diabetes, gender‐affirming hormonal therapy, and hypothyroidism. Individuals planning lifestyle interventions to reduce weight, those taking weight loss medications or medications affecting ectopic lipids, or those undergoing bariatric surgery were not included in our analysis. When measured repeatedly, only baseline data were used for this analysis (Figure 1).

### MR measurements

All MR and metabolic examinations were performed after an overnight fasting period of at least 8 h.

#### Assessment of HCL content

Most measurements (13/16 studies measuring HCL) were performed using a 3T whole‐body scanner (Magnetom, Siemens Healthineers, Erlangen, Germany). HCL was measured using ^1^H single‐voxel spectroscopy sequence (point‐resolved spectroscopy [PRESS] echo time [TE] = 30 ms or stimulated echo acquisition mode [STEAM] TE = 10 ms), as described previously [12, 16, 22]. Three studies (*n* = 55) [15, 19, 20] were performed on a 7T whole‐body scanner (Magnetom) using a double‐tuned (^31^P/^1^H) surface coil (RAPID Biomedical GmbH, Rimpar, Germany) [20] and STEAM sequence with TE = 6 ms. Cross validation of the 7T measurements and comparison with 3T measurements demonstrated high correlation among the values obtained with the 7T and 3T scanners that have been reported previously [23].

#### Assessment of MYCL content

MR examinations for MYCL (16 studies) took place on a 3T system with the same setup as for HCL measurements. Acquisitions were gated by electrocardiogram (ECG) and performed in breath‐hold. A PRESS sequence with TE = 30 ms or TE = 40 ms was used with a voxel placed in the interventricular septum. HCL and MYCL values were expressed as a fat fraction percentage after correction for relaxation times [9, 11, 24].

#### Cardiac function analysis

Cardiac function MR was performed in nine studies on the same 3T system with the same setup as for MYCL measurements. Visualization of cardiac function was performed as described elsewhere [25]. A short‐axis cine series was used to quantify left ventricular function (end‐diastolic and end‐systolic volume [EDV and ESV], stroke volume [SV], ejection fraction [EF], and myocardial mass) in syngo.via imaging software (Siemens Healthineers).

The online Supporting Information provides detailed descriptions of MR methods.

### Anthropometrics and laboratory assessments

Venous blood sampling was performed between 7 a.m. and 9 a.m. after an overnight fast of at least 8 h, followed by a 2‐h oral glucose tolerance test with an ingestion of 75 g of glucose in most participants. At baseline, laboratory assessments included glucometabolic parameters (fasting glucose, fasting insulin, and hemoglobin A1c [HbA1c]), lipid parameters (triglycerides [TG], total cholesterol, high‐density lipoprotein [HDL] cholesterol, and low‐density lipoprotein [LDL] cholesterol), liver function parameters (aspartate aminotransferase, alanine aminotransferase, and γ‐glutamyltransferase), inflammatory parameters (high‐sensitivity C‐reactive protein), and B‐type natriuretic peptide. During the oral glucose tolerance test, glucose and insulin were assessed 120 min after glucose ingestion. Laboratory samples were collected, centrifuged, and immediately transferred to the central International Organization for Standardization (ISO)‐certified laboratory at the General Hospital Vienna for further processing (online Supporting Information). The homeostatic model assessment of insulin resistance index, as a surrogate estimate of hepatic insulin resistance, was calculated as follows: (fasting insulin [microunits per milliliter] × fasting glucose [milligrams per deciliter])/405. The Matsuda index was calculated to assess insulin sensitivity [26].

Medical history and current medication use were assessed in every participant. Patients with a medical history of T2DM or use of glucose‐lowering medications were diagnosed with diabetes. For other participants, diabetes was diagnosed according to the guidelines of the Austrian Diabetes Association based on international guidelines, with HbA1c ≥ 6.5% (48 mmol/mol), fasting glucose ≥ 126 mg/dL (7.0 mmol/L), and/or 2‐h postprandial glucose ≥ 200 mg/dL (11.1 mmol/L) [27]. PreDM was classified as HbA1c ≥ 5.7% (39 mmol/mol) and <6.5% (48 mmol/mol), fasting glucose ≥ 100 mg/dL (5.6 mmol/L) and <126 mg/dL (7.0 mmol/L), and/or 2‐h postprandial glucose ≥ 140 mg/dL (7.8 mmol/L) and <200 mg/dL (11.1 mmol/L) [27].

### Data processing

Data were processed in Microsoft Excel (Microsoft Office Professional Plus 2016, Microsoft Corp., Redmond, Washington) and SPSS software version 28 (IBM Corp., Armonk, New York). For further analysis, participants were divided into three groups based on glucose tolerance, i.e., NG, preDM, and T2DM, as described earlier.

Participants were also divided into two groups based on their BMI, creating a group without OB, i.e., the nOB group, with BMI < 30 kg/m^2^, and a group with OB, i.e., the OB group, with BMI  ≥ 30 kg/m^2^. In total, this approach resulted in six separate subgroups.

### Statistical analysis

In order to describe the data pool, metric data are described using mean (standard deviation [SD]) if normally distributed or by median (minimum; maximum) in the case of skewed metric data. Nominal data are described as absolute frequency. In order to compare sexes, BMI groups, and glucose tolerance groups regarding HCL and MYCL, cardiac function, SV, and left ventricle volumes, three‐way ANCOVA (using age as a covariate) with post hoc simple effects analyses was used. The resulting estimated marginal mean and standard error (SE) are used as descriptive statistics. Owing to skewed physiological parameters, Spearman rank correlation coefficients (rho) were calculated to describe the association between physiological parameters and ectopic lipids. *P* values ≤ 0.05 are considered statistically significant. In order to avoid an increased error of the second type, no multiplicity corrections were performed.

## RESULTS

The selected cohort consisted of 503 participants (260 female and 243 male individuals), covering an age range from 17 to 78 years and BMI values from 17.1 to 48.5 kg/m^2^. Regarding glucose metabolism, there were 302 participants with NG, 62 with preDM, and 137 with T2DM. A total of 453 participants presented with HCL, 436 presented with MYCL, and both parameters were present for 410 participants (Figure 1; Table 1).

**TABLE 1:** Basic description of the cohort.

	Female individuals	Male individuals	All
Count	260	243	503
NG	165	137	302
PreDM	32	30	62
T2DM	63	74	137
nOB	187	176	363
OB	73	67	140
Mean ± SD			
Age (y)	40.90 (16.44)	44.51 (15.60)	42.67 (16.10)
BMI (kg/m^2^)	26.95 (5.68)	27.67 (4.58)	27.31 (5.19)
Systolic blood pressure (mm Hg)	123.62 (18.69)	130.49 (16.91)	126.85 (19.19)
Diastolic blood pressure (mm Hg)	76.90 (11.45)	79.24 (12.00)	77.00 (11.75)
Total cholesterol (mg/dL)	190.78 (39.40)	188.09 (47.42)	189.51 (43.35)
LDL cholesterol (mg/dL)	110.33 (64.46)	109.17 (38.13)	109.78 (53.68)
HDL cholesterol (mg/dL)	60.98 (17.88)	48.61 (12.61)	55.18 (16.79)
TG (mg/dL)	116.32 (87.91)	166.11 (187.99)	140.02 (146.43)
HOMA‐IR	2.49 (2.61)	2.62 (2.61)	2.55 (2.61)
Matsuda index	7.21 (4.80)	6.89 (6.18)	7.05 (5.53)

*Note*: Data given as absolute frequency (NG, preDM, T2DM, nOB and OB) and mean (SD) (age; BMI; blood pressure; total, HDL, and LDL cholesterol; TG; HOMA‐IR; and Matsuda index). nOB indicates BMI < 30 kg/m^2^, and OB indicates BMI ≥ 30 kg/m^2^.

Abbreviations: HDL, high‐density lipoprotein; HOMA‐IR, homeostatic model assessment of insulin resistance; LDL low‐density lipoprotein; NG, normoglycemia; nOB, no obesity; OB, obesity; preDM, prediabetes; T2DM, type 2 diabetes mellitus; TG, triglycerides.

Cohort characteristics, divided into subgroups based on their glucose tolerance and BMI, are presented in online Supporting information.

### MYCL content

**FIGURE 2:**
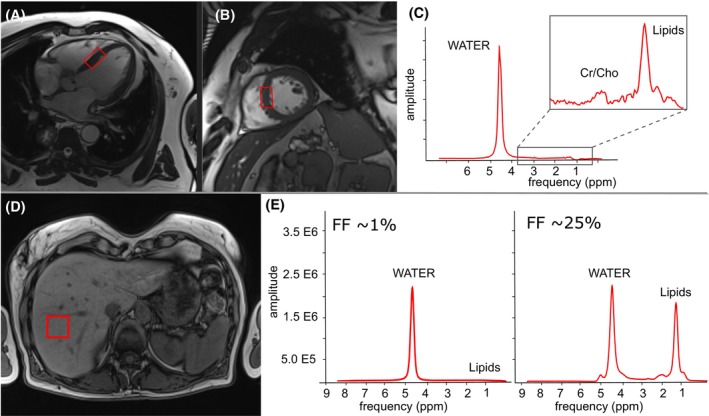
Imaging and spectroscopy of the heart and liver. CineTruffi images of (A) four‐chamber and (B) short‐axis view of the heart and position of the voxel in the intervertebral septum. (C) Example of the spectra with unsuppressed water and zoomed range with lipids and creatine and choline (Cr/Cho) signals. (D) Position of single‐voxel volume in the liver with resulting liver spectra with (E) low value fat fraction (FF) (~1%) and high value FF (~25%).

**FIGURE 3:**
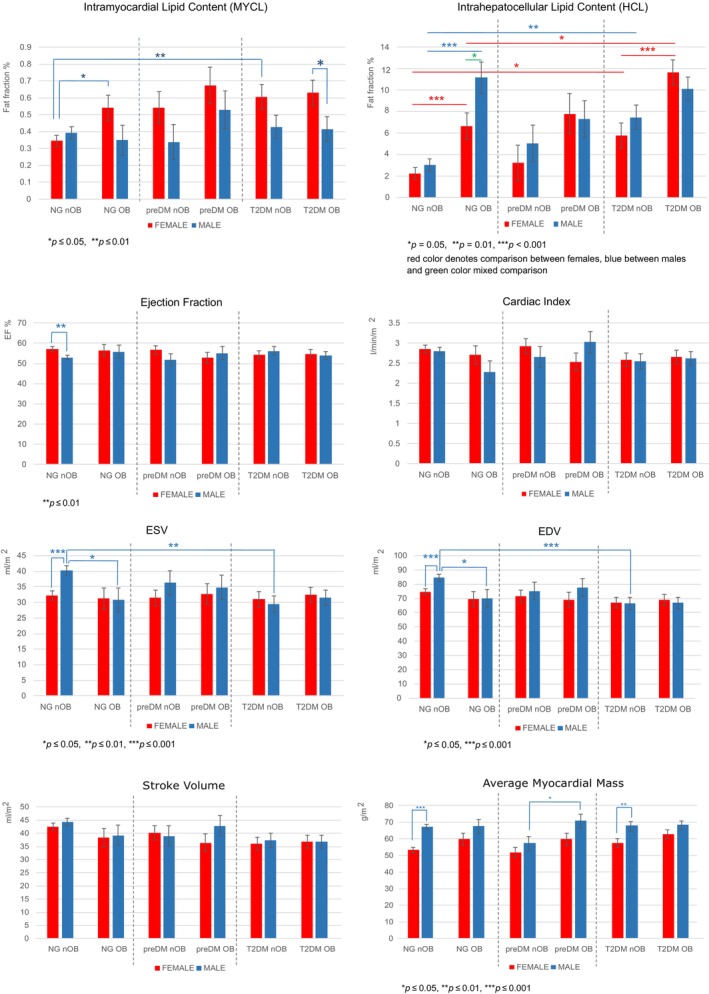
Lipid content in heart and liver and cardiac function parameters in male (blue) and female individuals (red) without obesity (nOB; BMI < 30 kg/m^2^) and with obesity (OB; BMI ≥ 30 kg/m^2^) with different grades of impairment of glucose metabolism. *p ≤ 0.05; **p ≤ 0.001; and ***p ≤ 0.001. Graphs are based on estimated marginal mean with SE. All values can be found in Table S4. ESV, end‐systolic volume; EDV, end‐diastolic volume; NG, normoglycemia; preDM, prediabetes; T2DM, type 2 diabetes.

The position of the voxel in the heart and an example of the spectra are shown in Figure 2A,B. Analysis showed a significant effect of sex and age on the amount of fat present in the myocardium, with higher levels in female than male individuals. The most prominent difference was present in the OB group with T2DM, with significantly higher fat percentages in female compared with male individuals (estimated marginal mean [SE]: 0.629% [0.078%] vs. 0.415% [0.074%]; *p* = 0.035; Figure 3).

For female individuals, in contrast to male individuals, BMI was an important determinant: female individuals with NG and OB had significantly higher MYCL than female individuals with NG and nOB (0.543% [0.075%] vs. 0.345% [0.034%]; *p* = 0.015). In nOB female individuals, T2DM was associated with significantly higher MYCL in comparison with those with NG (0.607% [0.071%] vs. 0.345% [0.034%]; *p* = 0.005).

### Cardiac function

**FIGURE 4:**
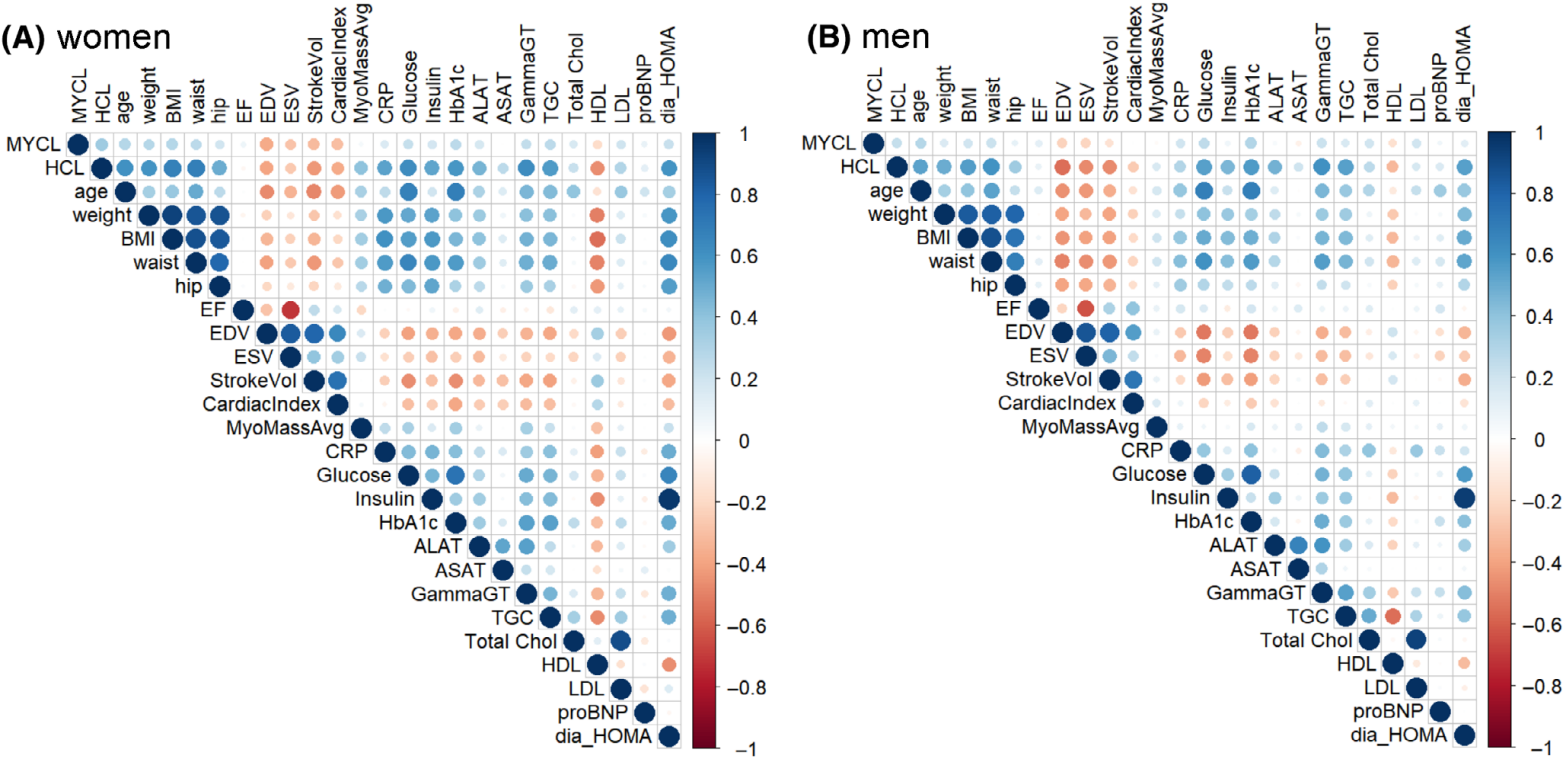
Correlation matrices for male and female individuals. Size of the circles as well as their color codes the strength of the correlation. Image created with the ggcorrplot package in R.

Although statistical analysis proved no effect of any studied parameter on EF, among nOB participants with NG, male individuals featured higher EF levels than female individuals (57.09% [1.19%] and 53.01% [1.15%]; *p* = 0.008; Figure 3). There was also moderate negative correlation of EF with ESV, both for male (−0.640; *p* < 0.001) and female individuals (−0.719; *p* < 0.001; Figure 4).

The cardiac index exhibited only a marginal dependency on age but an independency on any other studied parameter without any sex difference (Figure 3).

SV was affected only by age. There were no differences observed among the groups.

Analysis showed the dependency of the average myocardial mass of the ventricle on age, BMI, and sex separately, with no moderation effect. In general, female individuals showed smaller mass than male individuals (the value was normalized to body surface area). Within our groups, there was a significant difference between nOB female and male individuals with NG (53.25 [1.62] g/m^2^ vs. 66.97 [1.55] g/m^2^; *p* < 0.001) and also between nOB female and male individuals with T2DM (57.59 [2.68] g/m^2^ vs. 67.60 [2.94] g/m^2^; *p* = 0.009; Figure 3).

The volume of the left ventricle at the end of systole (ESV) was affected by age. Among participants with NG, we found higher volume in nOB male individuals in comparison with nOB female individuals (40.30 [1.42] mL/m^2^ vs. 32.18 [1.48] mL/m^2^; *p* < 0.001). nOB male individuals with NG had significantly higher ESV than male individuals with NG and OB (*p* = 0.021) and also significantly higher ESV than nOB male individuals with T2DM (29.43 [2.68] mL/m^2^; *p* = 0.002; Figure 3).

For left ventricular volume at the end of diastole (EDV), there was an effect of age and a marginal effect of glucose tolerance. It showed the same pattern as ESV, which means that nOB male individuals with NG had significantly larger EDV than nOB female individuals with NG (84.83 [2.26] mL/m^2^ vs. 74.63 [2.35] mL/m^2^, *p* = 0001), larger than male individuals with NG and OB (70.01 [6.32] mL/m^2^; *p* = 0.024) and larger than nOB male individuals with T2DM (66.69 [4.27] mL/m^2^; *p* = 0.001; Figure 3).

### HCL content

Age, glucose tolerance status, and BMI all affected the level of liver fat accumulation (i.e., HCL). Although sex did not affect it directly, there was a trend toward a moderating effect of glucose tolerance, BMI, and sex together (*p* = 0.053).

We observed increasing HCL for both sexes with increasing age (correlation 0.604 for female individuals, 0.535 for male individuals; both *p* < 0.001) and BMI (correlation 0.672 for female individuals, 0.559 for male individuals; both *p* < 0.001) and with decreasing glucose tolerance, which was more consistent in female individuals, whose HCL differs significantly with increasing BMI in both NG groups, as well as those with T2DM, whereas, in male individuals, higher HCL was only found in the NG group. There is a general trend of higher HCL in male individuals, with the most prominent difference between male and female individuals in the NG OB group (11.178% [1.476%] vs. 6.677% [1.233%]; *p* = 0.019). This trend changes in the OB T2DM group, in which female individuals tend to have slightly higher HCL than male individuals (Figure 3). An example of spectra from the liver is presented in Figure 2E.

Results of ANCOVA general linear model and estimates can be found in online Supporting information.

When looking at correlations between HCL and MYCL, only weak associations were observed in both sexes (0.234, *p* = 0.001 for male individuals and 0.356, *p* < 0.001 for female individuals).

Correlations of physiological parameters and HCL were very similar for both sexes, the strongest being γ‐glutamyltransferase (0.629 and 0.608 for female and male individuals, respectively; both *p* < 0.001), followed by homeostatic model assessment of insulin resistance, TG, and HbA1c. MYCL and cardiac function correlations showed no differences between the sexes. For correlations of cardiac function and physiological parameters, only a few differences between sexes were present, but those did not exceed a correlation coefficient of ±0.6, and intersex differences did not exceed 0.2. Correlation matrices are presented in Figure 4.

In this analysis, results are adjusted for age. In general, HCL and MYCL show weak to medium positive correlations with age in both sexes (female individuals: 0.288 [MYCL], 0.604 [HCL]; male individuals: 0.283 [MYCL], 0.535 [HCL]; *p* < 0.001 all). In HCL, the effect of age is also moderated by glucose tolerance status and BMI, but not by sex (details in online Supporting Information).

## DISCUSSION

Our results reveal increasing MYCL content with the progression from NG to T2DM, aggravated by important determinants such as increasing age and BMI only in female individuals, whereas, in male individuals, no such effects were seen with advancing impairment of glucose metabolism. HCL increased in both sexes with worsening of glucose metabolism and was also aggravated with OB and higher age, but this was more pronounced in female compared with male individuals. Furthermore, sex differences in cardiac remodeling and ventricular function were observed, which seem more severe in male individuals. This analysis gives us novel insight into sex‐specific differences in ectopic lipid accumulation in the heart and the liver, as well as cardiac function across different grades of impairment of glucose metabolism and further differentiation between individuals without and with OB.

We found similar MYCL in NG and nOB female compared with male individuals. However, significantly higher levels were found in female individuals with OB and T2DM compared with their male counterparts. Previous studies have highlighted higher MYCL in male compared with female individuals, which seems to change with deteriorating glucose metabolism from NG to T2DM [1, 8]. Another study investigating sex differences in MYCL in healthy adult volunteers with normal weight observed slightly higher levels in male individuals, which were not significantly different compared with female individuals and therefore are in accordance with our study [7]. No sex differences in MYCL at baseline were observed, and similar increases in male and female healthy participants with normal weight were demonstrated in a small experimental study during a hyperglycemic‐hyperinsulinemic clamp inducing short‐term hyperglycemia [25].

We observed a more pronounced increase of MYCL with a progression from NG to T2DM with significant differences between nOB female individuals with NG and nOB female individuals with T2DM. This is in line with an earlier study, which also reported a stronger increase of MYCL in female individuals with hyperglycemia (impaired glucose tolerance [IGT] or T2DM combined) [28]. Nonetheless, in this prior study, MYCL in male and female individuals with IGT or T2DM was not significantly different, and, due to small sample sizes, subgroups could not be analyzed in detail [28]. It was further speculated that these findings might be associated with the attenuation of cardiovascular protection and, consequently, the increase of cardiovascular risk observed in female individuals progressing to T2DM [8]. Another study investigating female individuals only but differentiating between different grades of impairment of glucose metabolism found significantly higher MYCL with the manifestation of T2DM and a trend of higher MYCL with IGT, but not in the group with insulin resistance compared with insulin‐sensitive female individuals [21]. This led to the conclusion that insulin resistance was not associated with cardiac steatosis. We also did not find associations of MYCL with insulin resistance, glucose, or HbA1c levels. Recently, Dong et al. [29] reported independent associations of insulin resistance, diabetes, and BMI with MYCL, as well as the highest levels of MYCL in participants with both diabetes and higher BMI, compared with healthy individuals. However, in their analysis, no differences between male and female individuals were observed. Likewise, Banerjee et al. reported no differences between male and female individuals in the associations of MYCL with OB [30], whereas other researchers have demonstrated associations of insulin resistance with MYCL in female individuals with OB [31]. In our observations, MYCL was comparable in NG and nOB male and female individuals; however, huge differences were seen in the population with OB. Female individuals with NG and OB had significantly higher MYCL compared with nOB female individuals with NG, whereas, in male individuals, no such differences were found between OB and nOB.

In line with prior studies [28], we also observed smaller myocardial ventricle mass in female individuals, with significant differences found between nOB female and male individuals with NG and nOB female and male individuals with T2DM. As described in a recently published review, several cross‐sectional studies have identified correlations between MYCL and cardiac function and concentric remodeling of the left ventricle [6]. Former studies have shown an impairment of left ventricular diastolic function in participants with T2DM compared with healthy individuals matched for BMI, age, and sex [32]. However, thus far, evidence is conflicting, and potential sex differences have not been well investigated. Mechanisms behind the impairment of ventricular dysfunction might be related to lipid intermediates accumulating in the heart with OB or impairment of glucose metabolism, causing cardiac lipotoxicity [6, 33]. We found higher ESV and EDV in nOB male individuals with NG compared with nOB female individuals with NG, as well as in nOB male individuals with NG compared with male individuals in NG and OB and nOB male individuals with T2DM. According to Maceira et al., the ESV and EDV values of nOB male and female individuals with NG are in the normal range [34]. Banerjee et al. [30] observed associations of MYCL with diastolic dysfunction and, in male individuals, only positive correlations of MYCL with concentric left ventricular remodeling. A more recent study also found concentric remodeling in both male and female individuals with T2DM but higher severity in male participants [35]. This is in contrast to another study reporting left ventricular concentric remodeling in female individuals with T2DM [36]. Additionally, this study also observed higher rates of diastolic dysfunction in male individuals with T2DM. No differences were found in EF or systolic or diastolic function between healthy controls and female individuals with T2DM, which is in line with our observations [36]. Worse left ventricular function in male individuals with T2DM compared with female individuals was also observed by Athithan et al. [35]. In female individuals with insulin resistance and T2DM, Krššák et al. [21] reported lower SV and heart rate but did not find any other differences in systolic and diastolic function. In conclusion from the aforementioned studies, the evidence regarding potential sex differences in the association of MYCL with cardiac remodeling and ventricular dysfunction is controversial and needs further investigation. Our investigations point to more severe structural and functional ventricular dysfunction in male individuals progressing to T2DM. OB and/or glucometabolic impairment are relevant risk factors for the development of heart failure with preserved EF in female individuals [1]. This might explain the less obvious changes in structural and functional ventricular parameters in female individuals observed in our analysis.

In both male and female individuals, we found higher HCL with increasing BMI, age, and impairment of glucose metabolism. These findings were confirmed by other studies that have also reported increases in HCL with OB or deterioration of glucose metabolism [5, 37]. Interestingly, an older study investigating sex differences in ectopic lipid accumulation found that higher age was associated with increased HCL in older female individuals, but not in male individuals [38]. This is in contrast to Schorr et al. [39], who found higher age‐ and BMI‐adjusted liver fat content in male compared with female individuals of the same age and BMI. Others have also observed significantly higher HCL in healthy male compared with female individuals when not considering BMI [5, 40]. Interestingly, this difference disappears between male and female individuals with worsening glucose metabolism, which is indicative of faster increases of HCL in female individuals progressing to T2DM [5, 40]. We found comparable HCL in male and female individuals with NG and nOB. However, with the deterioration of glucose metabolism, female individuals had higher increases in liver fat compared with male individuals, which was extraordinarily high in female individuals with OB. In both female individuals with NG and those with T2DM, we found significant differences in HCL between those in the OB and nOB groups, whereas, in male individuals, we can only observe these significant differences in the NG group. A recently published study corroborates our findings and also reported a higher risk of MASLD in female individuals with preDM and T2DM [4]. Additionally, in this study, a more pronounced impairment of metabolic risk factors due to worsening of glucose control was found in female compared to male individuals [4]. Moreover, Jesuthasan et al. recently suggested that a larger increase in very low‐density lipoprotein 1 (VLDL1)‐TG production rate in female individuals progressing to T2DM compared with male individuals and impairment of subcutaneous adipose tissue storage capacities might offer potential mechanisms involved in directing more lipids toward hepatic storage in female individuals [40].

Interestingly, in male individuals with OB, Mittendorfer et al. identified an increased VLDL‐triacylglycerol secretion compared with female individuals [41]. At similar BMI levels, male individuals have higher VLDL‐TG concentrations than female individuals [41], which are regulated to a larger extent by the VLDL‐TG secretion rate in male individuals and by the VLDL‐TG clearance rate in female individuals. Accordingly, in our analysis, the most prominent difference in HCL was found in male individuals with NG and OB, which was significantly different from female individuals with NG and OB, as well as from their leaner male counterparts. Former studies have shown higher capacities of gluteofemoral and subcutaneous lipid storage in healthy female individuals, which are considered to be associated with a lower risk of cardiometabolic disease and the release of metabolically beneficial adipokines [1]. A higher accumulation of lipids in subcutaneous adipose tissue, as well as a high expression of lipoprotein lipase, was suggested to be associated with higher VLDL1‐TG uptake [40]. These features and surrogate markers might be involved in the sex‐specific differences seen in cardiovascular outcomes in individuals progressing to T2DM and the attenuation of protective effects in female individuals with impairment of glucose metabolism or OB. Furthermore, sex‐specific associations of HCL content with different cardiovascular risk factors might also be relevant determinants contributing to sex differences in cardiovascular outcomes [4, 39].

This study has several limitations. Given the cross‐sectional nature, we cannot infer causality in relation to the observed structural and functional cardiac and hepatic sex differences. Because this cohort of participants consists of several different studies, not every study conducted the same assessments, and some parameters are missing. Therefore, 2‐h postprandial glucose was not available in all participants, and, in this case, we needed to base the diagnosis of preDM and T2DM on fasting glucose and HbA1c levels only. However, all studies were performed at the same institutions, and, thus, study protocols mostly used the same methodology and assessments. Patient numbers in the groups with preDM are low, and our differentiation into male and female nOB and OB subgroups made participant numbers per group even smaller. Owing to small sample sizes in subgroups, we were not able to analyze a participant group with normal weight with BMI < 25 kg/m^2^. However, such an analysis has not been performed so far, to our knowledge, and gives new insight into the effects of glucometabolic deteriorations and adiposity on ectopic lipid accumulation and cardiac function in male and female individuals. Moreover, glucose‐lowering treatment and/or lifestyle effects might be a reason for comparable HCL in participants with OB and T2DM and participants with NG and OB. Also, MYCL in the group with T2DM might be affected by lifestyle or background treatment compared with the groups with preDM. This analysis was conducted predominately in individuals of Caucasian ethnicity; therefore, results cannot be generalized.

## CONCLUSION

In conclusion, we found sex differences in MYCL and HCL, with more pronounced increases in MYCL and HCL in female individuals with progression from NG to T2DM compared with male individuals, aggravated by other important determinants such as increasing age and BMI. Furthermore, sex differences were found in cardiac remodeling and left ventricular dysfunction, with male individuals more affected with progression to T2DM. Understanding the sex differences in HCL and MYCL deposition is crucial for developing personalized treatment strategies for individuals with different stages of glucometabolic derangement. Further research is needed to understand these differences fully and to develop more effective, individualized treatment strategies.

## AUTHOR CONTRIBUTIONS

Jürgen Harreiter and Ivica Just researched data, contributed to the discussion, wrote the first draft of the manuscript, and reviewed and edited the manuscript. Michael Weber performed statistical analysis and reviewed and edited the manuscript. Ivica Just, Radka Klepochová, Marek Chmelík, and Martin Krššák performed MRI and analyzed MRI data. Radka Klepochová and Magdalena Bastian researched data and reviewed and edited the manuscript. Marek Chmelík, Yvonne Winhofer, Peter Wolf, Thomas Scherer, Michael Leutner, Lena Kosi‐Trebotic, Carol Deischinger, Michael R. Krebs, and Siegfried Trattnig reviewed and edited the manuscript. Martin Krššák and Alexandra Kautzky‐Willer contributed to the discussion and reviewed and edited the manuscript. All authors approved the final version of the manuscript.

## FUNDING INFORMATION

The studies included in this cohort analysis were funded by the Austrian Science Fund (FWF‐KLI 904B and FWF‐KLI 1122B to Martin Krššák), Austrian National Bank (ÖNB Jubiläumsfond 13249 and 15363 to Martin Krššák), and a European Foundation for the Study of Diabetes (EFSD) New Horizons Research Initiative grant. Alexandra Kautzky‐Willer and Jürgen Harreiter received funding for an investigator‐initiated study by AstraZeneca plc, project number ESR 15‐10882. The funders had no role in study design, data collection and analysis, decision to publish, or preparation of the manuscript.

## CONFLICT OF INTEREST STATEMENT

The authors declared no conflict of interest.

## CLINICAL TRIAL REGISTRATION

Clinical trial registration numbers of studies included in the cohort selection: ClinicalTrials.gov NCT01162772, NCT02023489, NCT02164032, NCT02075164, NCT01980524, NCT02023489, EudraCT 2007‐000353‐65, EudraCT 2016‐000574‐38, EudraCT 2017‐003014‐22.

## Supporting information


**Data S1.** Supporting Information.

**Data S2.** Supporting Information.

**Data S3.** Supporting Information.

**Data S4.** Supporting Information.

**Data S5.** Supporting Information.

## Data Availability

The datasets analyzed in the current study are available from the corresponding author upon reasonable request.
