# Supplementary material for: Sex differences in ectopic lipid deposits and cardiac function across a wide range of glycemic control: a secondary analysis
Source: Obesity (Silver Spring). 2024 Nov 18;32(12):2299–309. doi: 10.1002/oby.24153 (PMC11589534; doi:10.1002/oby.24153)
Supplement: Supplementary file 1 — Data S1. Supporting Information. [file OBY-32-2299-s003.pdf]

Supplementary Material 1

Full title: Sex differences in ectopic lipid deposits and cardiac function across a wide range of glycemic control: A secondary analysis

Authors: Jürgen Harreiter, PhD [1,2]*, Ivica Just, PhD [1,3]*, Michael Weber, PhD [4], Radka Klepochová, PhD[1,3], Magdalena Bastian, BSc [1], Yvonne Winhofer, PhD [1], Peter Wolf, PhD [1], Thomas Scherer, PhD [1], Michael Leutner, PhD [1], Lana Kosi-Trebotic, MD [1], Carola Deischinger, PhD [1], Marek Chmelík, PhD [3,5], Michael R Krebs, MD [1], Siegfried Trattnig, MD [3], Martin Krššák, PhD [1,3,#], Alexandra Kautzky-Willer, MD [1]

[1] Division of Endocrinology and Metabolism, Department of Internal Medicine III, Medical University of Vienna, Austria
[2] Department of Medicine, Landesklinikum Scheibbs, Austria
[3] High Field MR Center, Department of Biomedical Imaging and Image-guided Therapy, Medical University of Vienna, Austria
[4] Department of Biomedical Imaging and Image-guided Therapy, Medical University of Vienna, Austria
[5] Department of Technical Disciplines in Health Care at Faculty of Health Care, University of Prešov, Slovakia

* shared first authorship # - correspondent author

Correspondence and reprint requests: Martin Krššák, PhD, Department of Internal Medicine III, Medical University in Vienna, Währinger Gürtel 18-20
1090 Vienna, Austria E-Mail: martin.krssak@meduniwien.ac.at

Magnetic resonance measurements

Table S1-1. Spectroscopy methods overview, according to reporting standards by Lin et al (1)

Site (Name or Number)	High Field MR Centre, Medical University of Vienna		
	Heart (MYCL)	Liver (HCL)	Liver (HCL)
1. Hardware			
a. Field strength [T]	*3T*	*3T*	*7T*
b. Manufacturer	*Siemens*	*Siemens*	*Siemens*
c. Model (software version if available)	*Tim Trio, Prisma Fit**	*Tim Trio, Prisma Fit**	*Magnetom*
d. RF coils: nuclei (transmit/ receive), number of channels, type, body part	*18 channels matrix flex coil (Siemens)*<br><br>*32 array spine coil (Siemens)*	*18 channels matrix flex coil (Siemens)*<br><br>*32 array spine coil (Siemens)*	*double-tuned ($^{31}P/^{1}H$) surface coil with a diameter of 10 cm (Rapid Biomedical, Rimpar, Germany)*
e. Additional hardware	*N/A*	*N/A*	*N/A*
2. Acquisition			
a. Pulse sequence	*PRESS*	*PRESS, STEAM*	*STEAM*
b. Volume of Interest (VOI) locations	*Interventricular septum*	*Right lateral liver lobe*	*Right lateral liver lobe*
c. Nominal VOI size [cm^3, mm^3]	*15x15x15mm^3-15x15x35mm^3*	*30x30x20mm^3-30x30x30mm^3*	*30x30x30mm^3*
d. Repetition Time (TR), Echo Time (TE) [ms, s]	*TR variable depending on cardiac cycle*<br><br>*Tim Trio TE=30 ms*<br>*Prisma Fit TE=40ms*	*Tim Trio STEAM TR=2 s TE=10 ms*<br><br>*PrismaFit PRESS TR=2s TE=30/33 ms*	*TR=5s*<br><br>*TE=6ms*

e. Total number of Excitations or acquisitions per spectrum<br><br>In time series for kinetic studies<br>i. Number of Averaged spectra (NA) per time-point<br>ii. Averaging method (e.g. block-wise or moving average)<br>iii. Total number of spectra (acquired / in time-series)	*NA=4-8*<br><br>*Repetitions of acquisitions: 2*	*NA=4*<br><br>*Repetition of acquisition: 2-4*	*NA=8*
f. Additional sequence parameters<br><br>(spectral width in Hz, number of spectral points, frequency offsets)<br><br>If STEAM:, Mixing Time (TM)<br><br>If MRSI: 2D or 3D, FOV in all directions, matrix size, acceleration factors, sampling method	*-3.4ppm for WS spectra*<br><br>*0ppm for water reference*<br><br>*-2.3ppm for noWS spectra*	*-3.4ppm for WS spectra*<br><br>*0ppm for water reference*<br><br>*-2.3ppm for noWS spectra*<br><br>*STEAM TM=10ms*	*-3.4ppm for metabolite spectra*<br><br>*0ppm for water reference*<br><br>*STEAM TM=10ms*
g. Water Suppression Method	*WEAK water suppression, 50 Hz*	*Water saturation 35-55Hz*	*HLSVD in post-processing*
h. Shimming Method, reference peak, and thresholds for "acceptance of shim" chosen	*GRE shim & manual adjustments linewidth < 60Hz*	*GRE shim & manual adjustments linewidth < 70Hz*	*GRE shim & manual adjustments <80 Hz*
i. Triggering or motion correction method<br><br>(respiratory, peripheral, cardiac triggering, incl. device used and delays)	*ECG triggering*<br><br>*Breath-hold*	*Breath-hold*	*Free breathing*
3. Data analysis methods and outputs			
a. Analysis software	*spectroscopy processing tool provided by the system manufacturer (Siemens)*		*jMRUI software package version 5.0 (34) with the Advanced Method for Accurate, Robust and Efficient Spectral fitting (AMARES)*

b. Processing steps deviating from quoted reference or product			
c. Output measure<br><br>(e.g. absolute concentration, institutional units, ratio)Processing steps deviating from quoted reference or product	*fat fraction (FF) percentage ratio between lipids and the sum of water and lipid integrals*		
	Signals corrected for T_1 and T_2 relaxation based on measured values (2)	*signals, corrected for T_1 and T_2 relaxation based on measured values (3)*	*signals corrected for T_1 and T_2 relaxation based on measured values (4)*
d. Quantification references and assumptions, fitting model assumptions	*Prior knowledge of lipid peaks at 0.9ppm, 1.3ppm and water, 4.7ppm, with frequency constraints.*		
**4. Data Quality**			
a. Reported variables<br><br>(SNR, Linewidth (with reference peaks))	*Not reported*		
b. Data exclusion criteria	*FF>2% due to extra-cardiac fat contamination (5 subjects)*	*Not reported*	*Not reported*
c. Quality measures of postprocessing Model fitting (e.g. CRLB, goodness of fit, SD of residual)	*No QA measures described*		
d. Sample Spectrum	*Fig. 2*		

*3-Tesla whole body scanner was upgraded during the time period from TimTrio to Prisma Fit.

Cardiac function analysis. Heart MR examination for cardiac function was done in 9 studies on the same 3-Tesla system (Tim Trio & Prisma Fit, Siemens Healthineers, Erlangen, Germany) in the same session, patient position and coil combination as MYCL measurements, using ECG-triggering and acquired in breath-hold. Visualisation of cardiac function was performed using retrospective ECG-gated cine true fast imaging with steady-state precession (TrueFISP) sequences in two-chamber, four-chamber and short axes orientation. Short axes cine series (typically 10-12 slices, resolution $1.4 \times 2.1 \times 7.0$ mm^3, acquisition time per segment 48 ms, 25 reconstructed cardiac phases) were used to

quantify left ventricular global function (end-diastolic and end-systolic volume – EDV and ESV, stroke volume - SV, ejection fraction – EF and myocardial mass) via syngo.via. (Siemens Healthineers, Erlangen, Germany) by semiautomatic contouring endo- and epicardial borders in end-systolic and end-diastolic short axes cine images of the left ventricle. Papillary muscles were counted as muscle mass, and data were normalized to body surface area using the Dubois formula (body surface area = $0.007184 \times \text{height}^{0.725} \times \text{weight}^{0.425}$).

Anthropometrics and laboratory assessment

Plasma glucose was measured colorimetrically using hexokinase method (coefficient of variation (CV) 1.3%, glucose 101mg/dl), insulin by chemiluminescent immunoassay (sensitivity 2mIU/ml, CV 4-7%), HbA1c by high performance liquid chromatography (CV 1.8%, HbA1c 5.6%), triglycerides (CV=1.3% (125 mg/dl)), total cholesterol (CV=1.4% (101 mg/dL)), HDL cholesterol by enzymatic colorimetric methods. LDL cholesterol was calculated using Friedewald formula (triglycerides – HDL-C – (triglycerides/5)). The liver function parameters ALT, AST and GGT were assessed by enzyme kinetic testing with CVs of 2.2% (23U/L), 1.7% (17U/L) and 2.3% (26U/L) accordingly, hsCRP and NT-proBNP by immunological test (CV 2.7% (1.2mg/dl) and CV 3.7%). Details on the used laboratory methods are available on the homepage of the institute of laboratory medicine, www.kimcl.at.

Body height was measured to the nearest 0.1cm using a stadiometer. Body weight was measured to the nearest 0.1kg on calibrated electronic scales with the subjects wearing no shoes and lightly dressed. BMI was calculated as weight in kilograms divided by height in meters squared. Waist and hip circumferences were measured standing upright with the face directed toward the observer around the bare abdomen at the level midway between the lateral lower rib margin and the iliac crest and at the level of the widest circumference over the great trochanters, through the pubic symphysis. The waist/hip ratio was calculated as waist circumference divided by hip circumference.

Ethics numbers of studies included in the cohort selection

Ethical committee of Medical University in Vienna and Medical University in Graz

172/2002, 18-252 ex 06/07, 575/2008, 93/2010, 100/2010, 125/2011, 1310/2011, 1662/2012,

1022/2013,1128/2013, 2024/2013, 2197/2015, 1306/2016, 1629/2017, 1884/2017.

References

1. Lin A, Andronesi O, Bogner W, Choi IY, Coello E, Cudalbu C, Juchem C, Kemp GJ, Kreis R, Krssak M, Lee P, Maudsley AA, Meyerspeer M, Mlynarik V, Near J, Oz G, Peek AL, Puts NA, Ratai EM, Tkac I, Mullins PG, Experts' Working Group on Reporting Standards for MRS. Minimum Reporting Standards for in vivo Magnetic Resonance Spectroscopy (MRSinMRS): Experts' consensus recommendations. NMR Biomed 2021;34:e4484

2. Krssak M, Mlynarik V, Meyerspeer M, Moser E, Roden M. 1H NMR relaxation times of skeletal muscle metabolites at 3 T. MAGMA 2004;16:155-159

3. Winhofer Y, Krssak M, Wolf P, Tura A, Anderwald CH, Kosi L, Reiter G, Pacini G, Trattnig S, Luger A, Krebs M, Kautzky-Willer A. Hepatic rather than cardiac steatosis relates to glucose intolerance in women with prior gestational diabetes. PLoS One 2014;9:e91607

4. Gajdosik M, Chadzynski GL, Hangel G, Mlynarik V, Chmelik M, Valkovic L, Bogner W, Pohmann R, Scheffler K, Trattnig S, Krssak M. Ultrashort-TE stimulated echo acquisition mode (STEAM) improves the quantification of lipids and fatty acid chain unsaturation in the human liver at 7 T. NMR Biomed 2015;28:1283-1293
