# Supplementary material for: Sex differences in ectopic lipid deposits and cardiac function across a wide range of glycemic control: a secondary analysis
Source: Obesity (Silver Spring). 2024 Nov 18;32(12):2299–309. doi: 10.1002/oby.24153 (PMC11589534; doi:10.1002/oby.24153)
Supplement: Supplementary file 2 — Data S2. Supporting Information. [file OBY-32-2299-s005.pdf]

Supplementary Material 2

Full title: Sex differences in ectopic lipid deposits and cardiac function across a wide range of glycemic control: A secondary analysis

Authors: Jürgen Harreiter, PhD [1,2]*, Ivica Just, PhD [1,3]*, Michael Weber, PhD [4], Radka Klepochová, PhD [1,3] , Magdalena Bastian, BSc [1], Yvonne Winhofer, PhD [1], Peter Wolf, PhD [1], Thomas Scherer, PhD [1], Michael Leutner, PhD [1], Lana Kosi-Trebotic, MD [1], Carola Deischinger, PhD [1], Marek Chmelík, PhD [3,5], Michael R Krebs, MD [1], Siegfried Trattnig, MD [3], Martin Krššák, PhD [1,3,#], Alexandra Kautzky-Willer, MD [1]

[1] Division of Endocrinology and Metabolism, Department of Internal Medicine III, Medical University of Vienna, Austria
[2] Department of Medicine, Landesklinikum Scheibbs, Austria
[3] High Field MR Center, Department of Biomedical Imaging and Image-guided Therapy, Medical University of Vienna, Austria
[4] Department of Biomedical Imaging and Image-guided Therapy, Medical University of Vienna, Austria
[5] Department of Technical Disciplines in Health Care at Faculty of Health Care, University of Prešov, Slovakia
* shared first authorship # - correspondent author

Correspondence and reprint requests: Martin Krššák, PhD, Department of Internal Medicine III, Medical University in Vienna, Währinger Gürtel 18-20, 1090 Vienna, Austria
E-Mail: martin.krssak@meduniwien.ac.at

Table S2. Description of the data pool, divided into studied groups, with means (±SD)
(NGT= normoglycemia, preDM=prediabetes, T2DM= type 2 diabetes, BP-blood pressure)

		BMI<30 (no obesity)			BMI≥30 (obesity)		
		NGT	preDM	T2DM	NGT	preDM	T2DM
**AGE [years]**	N [f/m]	**137/123**	**18/17**	**32/36**	**28/14**	**14/13**	**31/38**
women	260	32.25 ± 12.70	49.44 ± 12.68	54.19 ± 11.78	37.29 ± 16.46	52.50 ± 9.74	58.48 ± 10.68
men	243	35.22 ± 12.88	51.29 ± 11.63	56.33 ± 10.04	38.63 ± 15.05	53.46 ± 7.09	58.74 ± 7.56
**WEIGHT [kg]**		**128/114**	**18/17**	**32/36**	**26/12**	**14/13**	**31/38**
Women	249	65.20 ± 9.94	65.62 ± 8.58	71.67 ± 9.03	91.78 ± 11.04	95.45 ± 11.07	94.14 ± 10.78
Men	230	79.11 ± 10.05	85.24 ± 9.64	83.70 ± 10.70	105.41 ± 16.28	106.15 ± 11.59	103.21 ± 14.53
**HEIGHT [cm]**		**128/114**	**18/17**	**32/36**	**26/12**	**14/13**	**31/38**
women	249	166.5 ± 5.93	165.31 ± 5.01	163.30 ± 8.46	166.35 ± 7.22	165.14 ± 8.80	165.10 ± 7.25
men	230	178.39 ± 7.41	178.76 ± 6.26	176.35 ± 7.76	175.50 ± 8.57	177.35 ± 7.16	174.26± 10.05
**BP systolic [mmHg]**		**109/83**	**27/36**	**32/33**	**9/7**	**20/8**	**37/40**
women	234	116.31 ± 13.09	120.05 ± 22.73	129.34 ± 19.37	131.33 ± 17.56	123.18 ± 16.39	141 .16 ± 17.60
men	207	126.74 ± 13.71	129.62 ± 14.14	135.85 ± 25.81	137.29 ± 16.33	131.00 ± 18.38	133.38 ± 14.72
**BP diastolic [mmHg]**		**109/83**	**27/36**	**32/33**	**9/7**	**20/8**	**37/40**
women	234	73.27 ± 10.89	72.83 ± 9.69	79.34 ± 8.90	87.11 ± 8.02	81.50 ± 12.37	83.51 ± 10.97
men	207	75.09 ± 10.82	79.24 ± 10.98	85.15 ± 11.93	86.57 ± 13.59	83.75 ± 16.36	80.78 ± 11.40
**GLUCOSE [mg/dL]**		**132/123**	**17/17**	**20/28**	**26/16**	**14/13**	**27/33**
women	236	80.52 ± 7.84	94.41 ± 11.41	140.90 ± 36.46	87.92 ± 8.81	100.14 ± 9.42	161.78 ± 51.96
men	230	84.78 ± 7.07	100.35 ± 9.06	155.32 ± 59.36	88.06 ± 6.83	100.54 ± 9.58	157.30 ± 41.10
**INSULIN [µlU/mL]**		**130/118**	**17/17**	**17/23**	**25/14**	**14/13**	**17/27**
women	220	7.03 ± 4.03	10.51 ± 5.37	11.11 ± 8.74	11.26 ± 5.31	21.11 ± 9.68	19.18 ± 12.72
men	212	7.49 ± 3.73	7.79 ± 4.02	11.14 ± 6.09	11.44 ± 4.84	16.49 ± 7.63	16.05 ± 11.78
**MATSUDA**		**89/81**	**25/29**	**2/7**	**6/8**	**18/8**	**9/17**
women	149	9.27 ± 4.47	4.12 ± 1.82	1.95 ± 0.35	8.47 ± 5.08	3.06 ± 0.73	4.03 ± 6.57
men	150	9.72 ± 7.09	4.07 ± 1.23	2.61 ± 1.56	6.49 ± 2.87	2.70 ± 0.35	2.15 ± 1.20
**HOMA**		**115/94**	**28/39**	**21/24**	**10/8**	**21/12**	**25/33**
women	220	1.14 ± 0.46	2.81 ± 0.80	3.81 ± 2.76	1.36 ± 0.56	3.11 ± 0.86	7.16 ± 4.40
men	210	1.19 ± 0.52	2.65 ± 0.59	3.75 ± 2.39	1.45 ± 0.47	3.08 ± 0.76	5.95 ± 4.48
**ASAT [U/L]**		**114/115**	**13/17**	**20/26**	**18/13**	**12/13**	**27/32**
women	204	23.11 ± 7.62	20.62 ± 4.82	21.35 ± 7.65	23.83 ± 7.15	24.67 ± 7.55	30.19 ± 15.85
men	216	28.17 ± 11.62	27.06 ± 8.39	33.42 ± 15.70	35.15 ± 19.40	28.77 ± 6.97	27.78 ± 11.49
**ALAT [U/L]**		**144/117**	**13/17**	**20/26**	**21/13**	**12/13**	**27/31**
women	207	19.43 ± 7.14	19.77 ± 4.85	27.20 ± 16.35	25.33 ± 13.71	33.58 ± 18.38	34.89 ± 17.47
men	217	30.67 ± 18.58	33.82 ± 19.12	48.88 ± 29.71	60.08 ± 55.82	34.69 ± 17.09	52.35 ± 89.10
**GammaGT [U/L]**		**114/115**	**13/17**	**20/26**	**18/13**	**12/13**	**27/32**
women	204	14.20 ± 5.92	19.46 ± 13.53	27.15 ± 11.54	25.17 ± 31.28	40.25 ± 34.59	43.81 ± 29.85
men	216	26.21 ± 24.08	46.88 ± 40.18	102.62 ± 162.50	44.38 ± 29.48	47.15 ± 42.07	56.91 ± 44.42
**TGC [mg/dL]**		**137/122**	**18/17**	**26/26**	**28/16**	**14/13**	**27/33**
Women	250	87.55 ± 54.63	109.28 ± 44.64	193.50 ± 185.29	118.43 ± 57.83	148.07 ± 35.35	174.00 ± 75.02
Men	227	146.18 ± 214.20	252.47 ± 270.45	166.58 ± 87.76	138.69 ± 52.01	167.08 ± 144.24	207.85 ± 129.66
**Chol [mg/dL]**		**128/113**	**18/17**	**26/26**	**26/12**	**14/13**	**27/33**
Women	239	185.45 ± 35.40	216.39 ± 31.77	207.42 ± 51.01	178.12 ± 33.54	199.21 ± 31.21	190.78 ± 47.83
Men	214	184.15 ± 45.45	216.12 ± 46.38	189.65 ± 64.23	188.08 ± 40.39	195.31 ± 45.33	183.09 ± 39.83
**HDL-C [mg/dL]**		**137/119**	**18/16**	**26/25**	**28/16**	**14/13**	**27/32**
Women	250	68.72 ± 17.84	61.83 ± 13.11	52.65 ± 15.51	47.07 ± 10.18	48.07 ± 9.54	50.22 ± 9.84
Men	221	50.71 ± 13.11	51.75 ± 18.13	46.40 ± 7.80	44.69 ± 8.45	45.15 ± 14.84	44.34 ± 9.44
**LDL-C [mg/dL]**		**137/119**	**18/16**	**26/25**	**28/16**	**14/13**	**27/32**
Women	250	99.31 ± 32.69	132.71 ± 27.55	120.90 ± 34.08	107.78 ± 31.48	121.53 ± 29.03	137.96 ± 170.80
Men	221	108.05 ± 36.46	122.06 ± 29.97	109.84 ± 54.84	122.89 ± 37.20	120.60 ± 41.26	99.39 ± 35.18
**CRP [mg/dL]**		**95/54**	**11/9**	**19/20**	**18/6**	**11/11**	**26/30**
Women	180	0.17 ± 0.25	0.18 ± 0.19	0.48 ± 0.65	0.41 ± 0.28	0.64 ± 0.51	0.62 ± 0.50
Men	130	0.14 ± 0.15	0.27 ± 0.29	0.58 ± 1.60	0.17 ± 0.13	0.29 ± 0.26	0.39 ± 0.40
**proBNP [pg/mL]**		**42/83**	**12/16**	**14/21**	**7/9**	**8/13**	**22/29**
Women	105	60.69 ± 36.30	49.09 ± 50.50	98.37 ± 121.34	74.54 ± 55.12	54.85 ± 44.56	76.81 ± 60.15
Men	171	29.89 ± 26.02	31.06 ± 24.56	85.50 ± 135.55	46.53 ± 43.13	37.98 ± 33.93	83.91 ± 229.66
**HbA1c [%] ok**		**133/103**	**18/17**	**28/29**	**25/14**	**14/13**	**27/35**
women	245	5.04 ± 0.31	5.67 ± 0.46	8.37 ± 1.58	5.20 ± 0.25	5.61 ± 0.32	8.20 ± 1.77
men	211	5.07 ± 0.29	5.69 ± 0.39	7.92 ± 1.47	5.26 ± 0.22	5.69 ± 0.31	7.76 ± 1.40
**HLC [%]**		**125/120**	**13/12**	**28/30**	**23/16**	**10/12**	**30/34**
women	229	1.27 ± 2.27	3.61 ± 4.61	4.04 ± 7.43	6.34 ± 7.86	8.87 ± 4.54	13.19 ± 8.43
men	224	2.40 ± 3.45	5.79 ± 4.55	8.78 ± 8.73	10.87 ± 13.31	8.23 ± 9.88	11.72 ± 8.21
**MLC [%]**		**135/106**	**15/12**	**27/31**	**23/16**	**11/10**	**23/27**
Women	234	0.31 ± 0.26	0.57 ± 0.47	0.35 ± 0.62	0.53 ± 0.35	0.71 ± 0.42	0.68 ± 0.56
Men	202	0.37 ± 0.34	0.36 ± 0.14	0.47 ± 0.38	0.34 ± 0.23	0.56 ± 0.34	0.47 ± 0.32
**Ejection Fraction [%]**		**56/61**	**14/7**	**19/16**	**9/7**	**10/7**	**18/21**
women	126	56.58 ± 7.73	56.88 ± 6.91	54.91 ± 7.42	56.43 ± 8.53	53.42 ± 5.70	55.38 ± 10.09
men	119	52.51 ± 7.46	52.12 ± 8.29	56.86 ± 6.96	54.97 ± 8.92	55.47 ± 5.25	54.64 ± 13.08
**Stroke Volume [ml/m2]**		**56/61**	**14/7**	**19/16**	**9/7**	**10/7**	**18/21**

women	126	43.90 ± 9.08	39.55 ± 9.62	34.24 ± 8.76	38.69 ± 9.97	34.78 ± 3.59	35.05 ± 7.70
men	119	45.64 ± 10.17	37.96 ± 8.81	35.14 ± 9.80	41.55 ± 14.50	41.85 ± 11.61	34.45 ± 17.33
**ESV [ml/m2]**		**56/61**	**14/7**	**19/16**	**9/7**	**10/7**	**18/21**
women	126	33.84 ± 8.60	30.76 ± 10.26	28.90 ± 10.17	31.53 ± 10.11	30.81 ± 6.89	30.44 ± 14.81
men	119	41.94 ± 10.69	35.22 ± 10.52	27.02 ± 10.23	33.33 ± 11.23	33.68 ± 10.26	27.73 ± 11.36
**EDV [ml/m2]**		**56/61**	**14/7**	**19/16**	**9/7**	**10/7**	**18/21**
women	126	77.75 ± 13.83	70.30 ± 17.90	63.14 ± 16.85	70.21 ± 16.51	65.59 ± 8.61	65.32 ± 19.31
men	119	87.97 ± 15.78	73.18 ± 15.45	62.16 ± 17.80	74.89 ± 23.15	75.53 ± 20.58	61.70 ± 21.78
**CARDIAC INDEX [l/min/m2**		**56/61**	**14/7**	**19/16**	**9/7**	**10/7**	**18/21**
women	126	2.94 ± 0.70	2389 ± 0.95	2.48 ± 0.61	2.73 ± 0.60	2.44 ± 0.37	2.55 ± 0.62
men	119	2.88 ± 0.67	2.60 ± 0.76	2.43 ± 0.73	2.42 ± 0.77	2.97 ± 0.79	2.48 ± 0.86
**Mass Average [g/m2]**		**56/61**	**14/7**	**19/16**	**9/7**	**10/7**	**18/21**
women	126	51.93 ± 8.86	52.50 ± 7.46	59.23 ± 7.65	59.55 ± 11.46	61.30 ± 5.88	64.47± 13.63
men	119	65.67 ± 13.00	58.10 ± 5.71	69.52 ± 16.22	65.16 ± 10.08	71.44 ± 18.24	70.21 ± 11.01

Table S3. Description of the data pool, divided into studied groups, with medians (min; max)
(NGT= normoglycemia, preDM=prediabetes, T2DM= type 2 diabetes, BP-blood pressure)

		**BMI<30 (no obesity)**			**BMI≥30 (obesity)**		
		**NGT**	**preDM**	**T2DM**	**NGT**	**preDM**	**T2DM**
**AGE [years]**		**137/123**	**18/17**	**32/36**	**28/14**	**14/13**	**31/38**
women	260	28.00 (17.00;71.00)	53.50 (20.00;69.00)	54.00 (28.00;72.00)	31 (20.00; 69.00)	52.00 (31.00;63.00)	59.00 (29.00;78.00)
men	243	31.00 (19.00;67.00)	50.00 (25.00;76.00)	56.50 (31.00;75.00)	38.50 (18.00;66.00)	53.00 (45.00;67.00)	60.50 (42.00;72.00)
**WEIGHT [kg]**		128/114	18/17	32/36	26/12	14/13	31/38
Women	249	65.00 (43.70;98.00)	65.10 (51.20;78.40)	73.00 (51.00;91.40)	91.00 (78.00;130.00)	93.55 (78.00;118.00)	93.30 (71.00;118.30)
Men	230	80.00 (51.00;101.10)	88.00 (65.70;99.30)	84.00 (51.00;107.30)	100.10 (82.00;137.90)	102.20 (91.20;131.90)	101.65 (74.50;135.30)
**HEIGHT [cm]**		128/114	18/17	32/36	26/12	14/13	31/38
women	249	166.00 (150.00;183.00)	166.50 (153.00;173.00)	162.50 (141.00;185.00)	167.50 (151.50;178.00)	170.00 (149.00;176.00)	166.00 (150.00;180.00)
men	230	179.50 (158.00;197.00)	180.00 (170.00;189.00)	176.00 (155.00;193.00)	174.50 (160.00;190.00)	176.00 (168.00;190.00)	175.00 (151.00;190.00)
**BP systolic [mmHg]**		109/83	27/36	32/33	9/7	20/8	37/40
women	234	115.00 (92.00;16.00)	115.67 (100.00;222.00)	124.50 (105.00;170.00)	125.00 (110.00;166.00)	123.50 (100.00;166.00)	140.00 (107.00;203.00)
men	207	126.00 (97.00;126.00)	129.00 (94.00;154.00)	130.00 (96.00;230.00)	135.00 (120.00; 158.00)	129.50 (110.00;168.00)	130.00 (108.00;179.00)
**BP diastolic [mmHg]**		109/83	27/36	32/33	9/7	20/8	37/40
women	234	72.00 (46.00;101.00)	71.00 (59.66;100.00)	79.00 (60.00;101.00)	89.00 (76.00; 100.00)	80.50 (60.33;110.00)	84.00 (59.00;119.00)
men	207	75.00 (49.67;101.00)	77.00 (56.00;110.00)	85.15 (64.00;121.00)	87.00 (70.00;112.00)	80.00 (59.00;118.00)	80.00 (55.00;104.00)
**GLUCOSE [mg/dL]**		132/123	17/17	20/28	26/16	14/13	27/33
women	236	80.00 (61.00;101.00)	92.00 (77.00;120.00)	137.00 (87.00;216.00)	87.50 (71.00;109.00)	102.00 (82.00;114.00)	148.00 (88.00;283.00)
men	230	85.00 (52.00;103.00)	97.00 (85.00;121.00)	139.00 (90.00;360.00)	89.00 (79.00;104.00)	100.00 (85.00;119.00)	145.00 (89.00;260.00)
**INSULIN [µlU/mL]**		130/118	17/17	17/23	25/14	14/13	17/27
women	220	6.48 (0.65;25.50)	8.33 (4.80;23.30)	9.30 (2.00;43.10)	11.30 (1.83;27.28)	21.30 (7.90;43.83)	13.90 (9.42;59.00)
men	212	7.61 (0.20;17.49)	6.89 (2.00;17.50)	11.20 (2.00;25.80)	11.23 (2.34;18.87)	20.20 (4.18;29.08)	12.00 (1.80;40.88)
**MATSUDA**		**89/81**	**25/29**	**2/7**	**6/8**	**18/8**	**9/17**
women	149	8.50 (3.30; 34.20)	3.90 (1.40; 10.10)	1.95 (1.70;2.20)	6.60 (5.90;18.80)	3.10 (1.60;4.40)	2.20 (0.80;21.40)
men	150	7.80 (3.40; 53.50)	4.20 (1.50;6.20)	2.10 (1.30;5.90)	5.80 (3.00;11.80)	2.70 (2.30;3.20)	1.80 (0.80;4.40)
**HOMA**		**115/94**	**28/39**	**21/24**	**10/8**	**21/12**	**25/33**
women	220	1.17 (0.12;1.98)	2.41 (2.06;4.86)	2.99 (0.77;13.62)	1.55 (0.39;1.99)	3.02 (2.11;5.38)	5.97 (3.05;20.10)
men	210	1.23 (0.04;1.98)	2.55 (2.01; 4.75)	3.22 (0.59;9.67)	1.55 (0.55;1.98)	3.01 (2.11;4.33)	5.27 (0.56;19.12)
**ASAT [U/L]**		114/115	13/17	20/26	18/13	41609	27/32
women	204	22 (9.00;70.00)	20.00 (13.00;32.00)	20 (12.00;49.00)	22.50 (17.00;47.00)	22.00 (16.00;43.00)	26.00 (18.00;98.00)
men	216	25.00 (15.00;102.00)	24.00 (19.00;51.00)	29.50 (16.00;68.00)	26.00 (23.00;89.00)	29.00 (18.00;40.00)	24.00 (15.00;61.00)
**ALAT [U/L]**		144/117	13/17	20/26	21/13	41609	27/31
women	207	18.00 (8.00;42.00)	19.00 (12.00;29.00)	21.5 (7.00;80.00)	21 (13.00;68.00)	29 (15.00;72.00)	27 (14.00;71.00)
men	217	28.00 (11.00;147.00)	28.00 (11.00;86.00)	38.50 (14.00;119.00)	32.00 (16.00;179.00)	30 (15.00;74.00)	31.00 (18.00;523.00)
**GammaGT [U/L]**		114/115	13/17	20/26	18/13	41609	27/32
women	204	13 (5.00;36.00)	13.00 (10.00;57.00)	25.5 (13.00;57.00)	15.5 (6.00;136.00)	32.50 (12.00;136.00)	34.00 (12.00;128.00)
men	216	19.00 (7.00;150.00)	29.00 (18.00;173.00)	34.00 (14.00;625.00)	33 (7.00;97.00)	42.00 (16.00;178.00)	39.50 (16.00;202.00)
**TGC [mg/dL]**		137/122	18/17	26/26	28/16	14/13	27/33
Women	250	78.00 (34.00;585.00)	98.00 (64.00;220.00)	137.50 (59.00;950.00)	93.50 (58.00;309.00)	147.00 (85.00;199.00)	149.00 (60.00;375.00)
Men	227	95.50 (33.00;1740.00)	148.00 (72.00;1096.00)	131.50 (61.00;377.00)	142.00 (31.00;231.00)	114.00 (70.00;601.00)	189.00 (73.00;778.00)
**Chol [mg/dL]**		128/113	18/17	26/26	26/12	14/13	27/33
Women	239	180.00 (113.00;298.00)	213.50 (163.00;297.00)	198.00 (133.00;354.00)	179.00 (123.00;239.00)	196.00 (154.00;274.00)	193.00 (133.00;322.00)
Men	214	177.00 (118.00;349.00)	210.00 (155.00;329.00)	184.50 (105.00;347.00)	190.50 (123.00;245.00)	198.00 (105.00;269.00)	172.00 (127.00;300.00)
**HDL-C [mg/dL]**		137/119	18/16	26/25	28/16	14/13	27/32
Women	250	67.00 (38.00;127.00)	62.50 (42.00;84.00)	51.50 (29.00;86.00)	45.00 (32.00;70.00)	46.50 (35.00;65.00)	49.00 (32.00;71.00)
Men	221	49 (6.00;91.00)	50.50 (29.00;104.00)	48.00 (27.00;64.00)	44.00 (34.00;67.00)	42.00 (28.00;79.00)	43 (29.00;74.00)
**LDL-C [mg/dL]**		137/119	18/16	26/25	28/16	14/13	27/32
Women	250	94.40 (39.00;190.00)	129.80 (86.60;191.80)	116.20 (73.00;206.40)	104.40 (50.80;162.00)	121.90 (76.20;187.20)	103.00 (50.40;966.00)
Men	221	103.00 (34.40;212.80)	116.50 (77.60;185.30)	111.80 (40.80;233.40)	127.60 (59.80;186.00)	126.80 (53.20;199.20)	91.90 (59.40;208.00)
**CRP [mg/dL]**		95/54	11/9	19/20	18/6	11/11	26/30
Women	180	0.07 (0.02;1.90)	0.09 (0.03;0.63)	0.24 (0.02;2.34)	0.37 (0.08;1.06)	0.51 (0.06;1.96)	0.36 (0.07;2.07)
Men	130	0.07 (0.02;0.67)	0.13 (0.06;0.90)	0.11 (0.03;7.25)	0.13 (0.04;0.36)	0.25 (0.03;0.86)	0.28 (0.03;1.67)
**proBNP [pg/mL]**		42/83	12/16	14/21	7/9	8/13	22/29
Women	105	54.30 (8.30;152.20)	37.50 (5.00;192.40)	55.20 (15.00;438.50)	50.4 (31.80;188.90)	41.45 (5.00;134.00)	60.6 (6.50;219.40)
Men	171	22.50 (2.50;130.70)	28.05 (5.00;111.30)	43.9 (5.00;634.40)	35.4 (5.00;149.10)	30.6 (5.00;139.20)	34.90 (5.00;1253.00)
**HbA1c [%] ok**		133/103	18/17	28/29	25/14	14/13	27/35
women	245	5.10 (4.00;5.70)	5.75 (4.70;6.20)	8.10 (6.50;12.00)	5.20 (4.80;5.60)	5.70 (4.90;6.00)	7.70 (5.70;11.70)
men	211	5.10 (4.30;5.60)	5.80 (5.00;6.20)	7.50 (5.70;11.50)	5.30 (4.80;5.60)	5.80 (5.00;6.20)	7.40 (5.80;11.50)
**HLC [%]**		**125/120**	**13/12**	**28/30**	**23/16**	**10/12**	**30/34**
women	229	0.57 (0.02;18.66)	1.43 (0.60;17.10)	3.83 (0.35;24.55)	2.81 (0.62;28.01)	9.38 (2.85;15.66)	11.40 (1.31;35.15)
men	224	1.09 (0.00;26.45)	5.98 (0.72;13.04)	5.39 (0.89;40.99)	4.10 (0.40;46.50)	4.80 (0.52;35.71)	9.38 (0.50;30.69)
**MLC [%]**		**135/106**	**15/12**	**27/31**	**23/16**	**11/10**	**23/27**
Women	234	0.25 (0.00;1.41)	0.33 (0.13;1.78)	0.46 (0.08;3.09)	0.42 (0.10;1.38)	0.58 (0.40;1.87)	0.35 (0.06;1.97)
Men	202	0.26 (0.01;1.60)	0.40 (0.11;0.54)	0.38 (0.10;1.67)	0.31 (0.01;0.85)	0.50 (0.17;1.46)	0.41 (0.01;1.60)
**Ejection Fraction [%]**		**56/61**	**14/7**	**19/16**	**9/7**	**10/7**	**18/21**
women	126	57.22 (24.24;69.46)	55.82 (48.04;68.01)	52.68 (41.69;70.72)	57.35 (44.92;69.79)	53.05 (46.71;63.47)	58.98 (36.99;70.55)
men	119	53.32 (36.90;67.85)	47.01 (45.52;64.17)	58.15 (41.92;65.04)	55.17 (43.35;68.77)	53.04 (51.19;64.60)	56.89 (20.70;76.66)
**Stroke Volume [ml/m2]**		**56/61**	**14/7**	**19/16**	**9/7**	**10/7**	**18/21**
women	126	43.86 (12.19;76.13)	36.68 (27.72;54.45)	34.55 (19.89;54.68)	37.85 (23.13;58.28)	35.19 (26.31;38.29)	35.05 (23.76;46.62)
men	119	45.52 (15.42;74.76)	37.16 (25.05;50.91)	36.01 (17.27;54.03)	40.62 (19.20;56.64)	39.38 (22.81;57.31)	32.32 (12.86;94.44)
**ESV [ml/m2]**		**56/61**	**14/7**	**19/16**	**9/7**	**10/7**	**18/21**
women	126	33.28 (14.28;58.06)	31.51 (14.38;51.54)	27.82 (11.82;47.76)	30.61 (17.68;46.07)	29.58(19.78;42.33)	28.19 (9.92;69.02)
men	119	40.80 (18.89;61.00)	34.85 (20.75;54.00)	25.55 (16.74;49.37)	33.73 (18.45;54.27)	34.87 (20.22;50.56)	27.8 (12.70;60.83)
**EDV [ml/m2]**		**56/61**	**14/7**	**19/16**	**9/7**	**10/7**	**18/21**
women	126	76.89 (36.68;123.39)	71.63 (42.32;103.55)	63.21 (39.09;93.19)	71.76 (40.81;95.60)	64.77 (54.14;79.43)	64.39 (33.69;109.53)
men	119	89.29 (35.71;134.62)	74.53 (54.62;99.98)	63.02 (35.67;103.40)	66.20 (44.29;110.91)	74.25 (44.56;103.79)	55.50 (38.04;123.20)
**CARDIAC INDEX [l/min/m2**		**56/61**	**14/7**	**19/16**	**9/7**	**10/7**	**18/21**
women	126	2.92 (0.78;5.74)	2.54 (2.15;5.03)	2.33 (1.49;3.94)	2.72 (1.80;3.79)	2.36 (2.03;3.20)	2.56 (1.71;3.64)
men	119	2.83 (1.33;4.75)	2.84 (1.28;3.72)	2.42 (1.19;3.60)	2.48 (1.34;3.40)	3.16 (1.46;3.73)	2.43 (0.85;4.91)
**Mass Average [g/m2]**		**56/61**	**14/7**	**19/16**	**9/7**	**10/7**	**18/21**
women	126	50.70 (34.35;73.16)	50.34 (44.20;62.41)	58.15 (42.04;73.90)	57.70 (44.87;82.57)	61.66 (50.22;69.77)	61.99 (45.60;93.29)
men	119	66.64 (2.13;103.16)	57.33 (47.96;65.77)	70.21 (31.70;101.21)	65.19 (48.13;80.56)	74.48 (43.25;87.63)	70.91 (50.37;88.84)
