# Supplementary material for: Sex differences in ectopic lipid deposits and cardiac function across a wide range of glycemic control: a secondary analysis
Source: Obesity (Silver Spring). 2024 Nov 18;32(12):2299–309. doi: 10.1002/oby.24153 (PMC11589534; doi:10.1002/oby.24153)
Supplement: Supplementary file 3 — Data S3. Supporting Information. [file OBY-32-2299-s002.pdf]

Supplementary Material 3

Full title: Sex differences in ectopic lipid deposits and cardiac function across a wide range of glycemic control: A secondary analysis

Authors: Jürgen Harreiter, PhD [1,2]*, Ivica Just, PhD [1,3]*, Michael Weber, PhD [4], Radka Klepochová, PhD [1,3], Magdalena Bastian, BSc [1], Yvonne Winhofer, PhD [1], Peter Wolf, PhD [1], Thomas Scherer, PhD [1], Michael Leutner, PhD[1], Lana Kosi-Trebotic, MD [1], Carola Deischinger, PhD [1], Marek Chmelík, PhD [3,5], Michael R Krebs, MD [1], Siegfried Trattnig, MD [3], Martin Krššák, PhD [1,3,#], Alexandra Kautzky-Willer, MD [1]

[1] Division of Endocrinology and Metabolism, Department of Internal Medicine III, Medical University of Vienna, Austria
[2] Department of Medicine, Landesklinikum Scheibbs, Austria
[3] High Field MR Center, Department of Biomedical Imaging and Image-guided Therapy, Medical University of Vienna, Austria
[4] Department of Biomedical Imaging and Image-guided Therapy, Medical University of Vienna, Austria
[5] Department of Technical Disciplines in Health Care at Faculty of Health Care, University of Prešov, Slovakia
* shared first authorship # - correspondent author

Correspondence and reprint requests: Martin Krššák, PhD, Department of Internal Medicine III, Medical University in Vienna, Währinger Gürtel 18-20, 1090 Vienna, Austria
E-Mail: martin.krssak@meduniwien.ac.at

Table S4. Estimated marginal means (Mean) and standard errors (SE) for all groups from ANCOVA general linear model.

OB – obesity with BMI≥30kgm^{-2}, nOB – non-obesity with BMI <30kgm^{-2}, NGT-normoglycaemia, preDM – pre-diabetes, T2DM –typ 2 diabetes.

MYCL - intramyocardial lipids

Glucose tolerance	BMI	Sex	Mean	SE	95% Confidence Interval Lower bound	95% Confidence Interval Upper bound
NGT	nOB	female	0,345[a]	0.034	0.278	0.411
		male	0,391[a]	0.036	0.321	0.461
	OB	female	0,543[a]	0.075	0.397	0.69
		male	0,350[a]	0.089	0.175	0.526
preDM	nOB	female	0,543[a]	0.093	0.36	0.725
		male	0,337[a]	0.104	0.133	0.541
	OB	female	0,672[a]	0.109	0.459	0.886
		male	0,529[a]	0.114	0.305	0.752
T2DM	nOB	female	0,607[a]	0.071	0.467	0.748
		male	0,428[a]	0.067	0.297	0.56
	OB	female	0,629[a]	0.078	0.476	0.782
		male	0,415[a]	0.074	0.27	0.559

a. The covariates in the model are calculated using the following values: age = 42.44.

HCL - intrahepatocellular lipids

Glucose tolerance	BMI	sex	Mean	SE	95% Confidence Interval Lower bound	95% Confidence Interval Upper bound
NGT	nOB	female	2,231[a]	0.585	1.081	3.381
		male	3,040[a]	0.564	1.932	4.147
	OB	female	6,677[a]	1.233	4.254	9.1
		male	11,178[a]	1.476	8.276	14.08
preDM	nOB	female	3,210[a]	1.639	-0.011	6.431
		male	5,046[a]	1.713	1.679	8.414
	OB	female	7,808[a]	1.885	4.103	11.513
		male	7,313[a]	1.719	3.935	10.692
T2DM	nOB	female	5,786[a]	1.162	3.503	8.069
		male	7,478[a]	1.13	5.257	9.699
	OB	female	11,667[a]	1.149	9.409	13.925
		male	10,138[a]	1.094	7.988	12.287

a. The covariates in the model are calculated using the following values: age = 42,09.

Cardiac Index						
Glucose tolerance	BMI	sex	Mean	SE	95% Confidence Interval	
					Lower bound	Upper bound
NGT	nOB	female	2,859[a]	0.101	2.66	3.058
		male	2,799[a]	0.097	2.608	2.99
	OB	female	2,708[a]	0.234	2.247	3.169
		male	2,283[a]	0.271	1.748	2.818
preDM	nOB	female	2,924[a]	0.188	2.553	3.294
		male	2,657[a]	0.266	2.132	3.181
	OB	female	2,537[a]	0.226	2.093	2.982
		male	3,029[a]	0.266	2.505	3.554
T2DM	nOB	female	2,580[a]	0.167	2.251	2.91
		male	2,549[a]	0.183	2.188	2.911
	OB	female	2,651[a]	0.171	2.314	2.988
		male	2,622[a]	0.165	2.298	2.946

a. The covariates in the model are calculated using the following values: age = 45,15.

Stroke Volume						
Glucose tolerance	BMI	sex	Mean	SE	95% Confidence Interval	
					Lower bound	Upper bound
NGT	nOB	female	42,438[a]	1.477	39.528	45.347
		male	44,203[a]	1.42	41.404	47.001
	OB	female	38,386[a]	3.425	31.637	45.135
		male	39,269[a]	3.974	31.439	47.099
preDM	nOB	female	40,168[a]	2.755	34.741	45.596
		male	38,935[a]	3.899	31.253	46.616
	OB	female	36,404[a]	3.304	29.895	42.913
		male	42,848[a]	3.9	35.165	50.531
T2DM	nOB	female	36,046[a]	2.451	31.218	40.874
		male	37,258[a]	2.686	31.965	42.551
	OB	female	36,796[a]	2.507	31.857	41.734
		male	36,834[a]	2.411	32.084	41.585

a. The covariates in the model are calculated using the following values: age = 45,15.

Ejection Fraction						
Glucose tolerance	BMI	sex	Mean	SE	95% Confidence Interval	
					Lower bound	Upper bound
NGT	nOB	female	57,088[a]	1.192	54.739	59.438
		male	53,013[a]	1.147	50.753	55.272
	OB	female	56,529[a]	2.766	51.08	61.979
		male	55,757[a]	3.209	49.435	62.08
preDM	nOB	female	56,668[a]	2.224	52.286	61.051
		male	51,782[a]	3.148	45.579	57.985
	OB	female	52,857[a]	2.668	47.601	58.113
		male	55,129[a]	3.149	48.925	61.333
T2DM	nOB	female	54,281[a]	1.979	50.382	58.18
		male	56,126[a]	2.169	51.852	60.4
	OB	female	54,773[a]	2.024	50.786	58.761
		male	53,809[a]	1.947	49.973	57.645

a.The covariates in the model are calculated using the following values: age = 45,15.

End of Diastole Volume						
Glucose tolerance	BMI	sex	Mean	SE	95% Confidence Interval	
					Lower bound	Upper bound
NGT	nOB	female	74,629[a]	2.348	70.003	79.255
		male	84,833[a]	2.259	80.382	89.283
	OB	female	69,571[a]	5.447	58.839	80.304
		male	70,005[a]	6.32	57.553	82.457
preDM	nOB	female	71,628[a]	4.381	62.997	80.258
		male	75,264[a]	6.2	63.048	87.479
	OB	female	69,056[a]	5.254	58.705	79.407
		male	77,661[a]	6.201	65.443	89.879
T2DM	nOB	female	66,996[a]	3.897	59.318	74.673
		male	66,694[a]	4.272	58.277	75.111
	OB	female	69,052[a]	3.986	61.199	76.905
		male	66,803[a]	3.834	59.248	74.357

a.The covariates in the model are calculated using the following values: age = 45,15.

End of Systole Volume						
Glucose tolerance	BMI	sex	Mean	SE	95% Confidence Interval	
					Lower bound	Upper bound
NGT	nOB	female	32,175[a]	1.473	29.274	35.077
		male	40,304[a]	1.417	37.512	43.095
	OB	female	31,186[a]	3.416	24.454	37.917
		male	30,739[a]	3.964	22.929	38.549
preDM	nOB	female	31,461[a]	2.748	26.048	36.874
		male	36,330[a]	3.889	28.668	43.991
	OB	female	32,650[a]	3.295	26.158	39.142
		male	34,812[a]	3.89	27.148	42.475
T2DM	nOB	female	30,949[a]	2.444	26.133	35.764
		male	29,431[a]	2.68	24.152	34.711
	OB	female	32,422[a]	2.5	27.496	37.347
		male	30,443[a]	2.405	25.705	35.181

a. The covariates in the model are calculated using the following values: age = 45,15.

Mass Average						
Glucose tolerance	BMI	sex	Mean	SE	95% Confidence Interval	
					Lower bound	Upper bound
NGT	nOB	female	53,249[a]	1.616	50.065	56.432
		male	66,971[a]	1.554	63.908	70.033
	OB	female	59,820[a]	3.748	52.435	67.206
		male	67,227[a]	4.349	58.658	75.796
preDM	nOB	female	51,937[a]	3.014	45.998	57.877
		male	57,221[a]	4.267	48.815	65.628
	OB	female	59,838[a]	3.615	52.715	66.961
		male	70,543[a]	4.267	62.135	78.951
T2DM	nOB	female	57,594[a]	2.682	52.311	62.877
		male	67,600[a]	2.94	61.807	73.392
	OB	female	62,897[a]	2.743	57.492	68.301
		male	68,058[a]	2.639	62.86	73.257

a. The covariates in the model are calculated using the following values: age = 45,15.
