# Supplementary material for: Sex differences in ectopic lipid deposits and cardiac function across a wide range of glycemic control: a secondary analysis
Source: Obesity (Silver Spring). 2024 Nov 18;32(12):2299–309. doi: 10.1002/oby.24153 (PMC11589534; doi:10.1002/oby.24153)
Supplement: Supplementary file 4 — Data S4. Supporting Information. [file OBY-32-2299-s001.pdf]

Supplementary Material 4

Full title: Sex differences in ectopic lipid deposits and cardiac function across a wide range of glycemic control: A secondary analysis

Authors: Jürgen Harreiter, PhD [1,2]*, Ivica Just, PhD [1,3]*, Michael Weber, PhD [4], Radka Klepochová[1,3], Magdalena Bastian, BSc [1], Yvonne Winhofer, PhD [1], Peter Wolf, PhD [1], Thomas Scherer, PhD [1], Michael Leutner, PhD[1], Lana Kosi-Trebotic, MD [1], Carola Deischinger, PhD [1], Marek Chmelík, PhD [3,5], Michael R Krebs, MD [1], Siegfried Trattnig, MD [3], Martin Krššák, PhD [1,3,#], Alexandra Kautzky-Willer, MD [1]

[1] Division of Endocrinology and Metabolism, Department of Internal Medicine III, Medical University of Vienna, Austria
[2] Department of Medicine, Landesklinikum Scheibbs, Austria
[3] High Field MR Center, Department of Biomedical Imaging and Image-guided Therapy, Medical University of Vienna, Austria
[4] Department of Biomedical Imaging and Image-guided Therapy, Medical University of Vienna, Austria
[5] Department of Technical Disciplines in Health Care at Faculty of Health Care, University of Prešov, Slovakia
* shared first authorship # - correspondent author

Correspondence and reprint requests: Martin Krššák, PhD, Department of Internal Medicine III, Medical University in Vienna, Währinger Gürtel 18-20, 1090 Vienna, Austria
E-Mail: martin.krssak@meduniwien.ac.at

Results of ANCOVA general linear model

MYCL

	Type III Sum of Squares	df	Mean Square	F	Sig.
age	0.593	1	0.593	4.663	0.031
glucose tolerance	0.634	2	0.317	2.491	0.084
BMI.30	0.383	1	0.383	3.010	0.083
sex	1.286	1	1.286	10.102	0.002
glucose tolerance * BMI.30	0.213	2	0.107	0.837	0.434
glucose tolerance * sex	0.244	2	0.122	0.958	0.385
BMI.30 * sex	0.074	1	0.074	0.578	0.448
glucose tolerance * BMI.30 * sex	0.260	2	0.130	1.020	0.362

a. R Squared = 0,119 (Adjusted R Squared = 0,094)

Pairwise comparisons

OUTCOME MYCL

BMI.30	sex	(I) glucose tolerance	(J) glucose tolerance	mean difference (I-J)	SE	Sig.[b]	95% Confidence Intervall for the lower bound	upper bound
BMI <30	female	normoglycemia	prediabetes	-0.198	0.100	0.147	-0.440	0.043
			type 2 diabetes	-0,263*	0.083	0.005	-0.461	-0.064
		prediabetes	normoglycemia	0.198	0.100	0.147	-0.043	0.440
			type 2 diabetes	-0.064	0.115	1.000	-0.341	0.212
		type 2 diabetes	normoglycemia	0,263*	0.083	0.005	0.064	0.461
			prediabetes	0.064	0.115	1.000	-0.212	0.341
	male	normoglycemia	prediabetes	0.054	0.110	1.000	-0.211	0.319
			type 2 diabetes	-0.037	0.078	1.000	-0.225	0.150
		prediabetes	normoglycemia	-0.054	0.110	1.000	-0.319	0.211
			type 2 diabetes	-0.091	0.122	1.000	-0.383	0.201
		type 2 diabetes	normoglycemia	0.037	0.078	1.000	-0.150	0.225
			prediabetes	0.091	0.122	1.000	-0.201	0.383
BMI 30+	female	normoglycemia	prediabetes	-0.129	0.132	0.989	-0.447	0.189
			type 2 diabetes	-0.086	0.109	1.000	-0.347	0.175
		prediabetes	normoglycemia	0.129	0.132	0.989	-0.189	0.447
			type 2 diabetes	0.043	0.131	1.000	-0.271	0.358
		type 2 diabetes	normoglycemia	0.086	0.109	1.000	-0.175	0.347
			prediabetes	-0.043	0.131	1.000	-0.358	0.271
	male	normoglycemia	prediabetes	-0.179	0.145	0.658	-0.527	0.170
			type 2 diabetes	-0.064	0.117	1.000	-0.345	0.217
		prediabetes	normoglycemia	0.179	0.145	0.658	-0.170	0.527
			type 2 diabetes	0.114	0.133	1.000	-0.204	0.433
		type 2 diabetes	normoglycemia	0.064	0.117	1.000	-0.217	0.345
			prediabetes	-0.114	0.133	1.000	-0.433	0.204

Based on estimated marginal means
*. The mean difference is significant at level 0.05.
b.Adjustment for multiple comparisons: Bonferroni.

Pairwise comparisons

OUTCOME MYCL

glucose tolerance	sex	(I) BMI.30	(J) BMI.30	mean difference (I-J)	SE	Sig.[b]	95% Confidence Interval for the lower bound	upper bound
normoglycemia	female	BMI <30	BMI 30+	-0,199*	0.081	0.015	-0.358	-0.039
	male	BMI <30	BMI 30+	0.041	0.096	0.671	-0.147	0.229
prediabetes	female	BMI <30	BMI 30+	-0.130	0.142	0.361	-0.408	0.149
	male	BMI <30	BMI 30+	-0.192	0.153	0.210	-0.492	0.108
type 2 diabetes	female	BMI <30	BMI 30+	-0.022	0.101	0.830	-0.221	0.177
	male	BMI <30	BMI 30+	0.014	0.094	0.884	-0.171	0.199

Based on estimated marginal means
*. The mean difference is significant at level 0.05.
b.Adjustment for multiple comparisons: Bonferroni.

Pairwise comparisons

OUTCOME MYCL

glucose tolerance	BMI.30	(I) sex	(J) sex	mean difference (I-J)	SE	Sig.[b]	95% Confidence Interval for the lower bound	upper bound
normoglycemia	BMI <30	female	male	-0.047	0.047	0.320	-0.138	0.045
	BMI 30+	female	male	0.193	0.116	0.098	-0.035	0.421
prediabetes	BMI <30	female	male	0.206	0.138	0.137	-0.066	0.477
	BMI 30+	female	male	0.143	0.156	0.358	-0.163	0.450
type 2 diabetes	BMI <30	female	male	0.179	0.094	0.058	-0.006	0.363
	BMI 30+	female	male	0,214*	0.101	0.035	0.015	0.413

Based on estimated marginal means
*. The mean difference is significant at level 0.05.
b.Adjustment for multiple comparisons: Bonferroni.

INTRAHEPATOCELLULAR LIPIDS

Tests of between-subjects effects

OUTCOME HCL

Source	Type III Sum of Squares	df	Mean Square	F	Sig.
age	504.089	1	504.089	14.498	0.000
glucose tolerance	472.626	2	236.313	6.796	0.001
BMI.30	1,287.545	1	1,287.545	37.030	0.000
sex	77.550	1	77.550	2.230	0.136
glucose tolerance * BMI.30	99.255	2	49.627	1.427	0.241
glucose tolerance * sex	109.642	2	54.821	1.577	0.208
BMI.30 * sex	5.772	1	5.772	0.166	0.684
glucose tolerance * BMI.30 * sex	205.850	2	102.925	2.960	0.053

a. R Squared = 0,345 (Adjusted R Squared = 0,327)

Pairwise comparisons

OUTCOME HCL

BMI.30	sex	(I) glucose tolerance	(J) glucose tolerance	mean difference (I-J)	SE	Sig.[b]	95% Confidence Interval for the lower bound	upper bound
BMI <30	female	normoglycemia	prediabetes	-0.979	1.755	1.000	-5.198	3.240
			type 2 diabetes	-3,555*	1.363	0.028	-6.831	-0.278
		prediabetes	normoglycemia	0.979	1.755	1.000	-3.240	5.198
			type 2 diabetes	-2.576	1.992	0.590	-7.362	2.210
		type 2 diabetes	normoglycemia	3,555*	1.363	0.028	0.278	6.831
			prediabetes	2.576	1.992	0.590	-2.210	7.362
	male	normoglycemia	prediabetes	-2.007	1.822	0.814	-6.385	2.372
			type 2 diabetes	-4,438*	1.307	0.002	-7.580	-1.296
		prediabetes	normoglycemia	2.007	1.822	0.814	-2.372	6.385
			type 2 diabetes	-2.432	2.019	0.688	-7.285	2.421
		type 2 diabetes	normoglycemia	4,438*	1.307	0.002	1.296	7.580
			prediabetes	2.432	2.019	0.688	-2.421	7.285
BMI 30+	female	normoglycemia	prediabetes	-1.131	2.264	1.000	-6.570	4.309
			type 2 diabetes	-4,990*	1.706	0.011	-9.090	-0.889
		prediabetes	normoglycemia	1.131	2.264	1.000	-4.309	6.570
			type 2 diabetes	-3.859	2.157	0.223	-9.042	1.324

		type 2 diabetes	normoglycemia	4,990*	1.706	0.011	0.889	9.090
			prediabetes	3.859	2.157	0.223	-1.324	9.042
	male	normoglycemia	prediabetes	3.865	2.275	0.270	-1.602	9.331
			type 2 diabetes	1.040	1.856	1.000	-3.419	5.499
		prediabetes	normoglycemia	-3.865	2.275	0.270	-9.331	1.602
			type 2 diabetes	-2.824	1.988	0.468	-7.601	1.952
		type 2 diabetes	normoglycemia	-1.040	1.856	1.000	-5.499	3.419
			prediabetes	2.824	1.988	0.468	-1.952	7.601

Based on estimated marginal means
*. The mean difference is significant at level 0.05.
b.Adjustment for multiple comparisons: Bonferroni.

Pairwise comparisons

OUTCOME HCL

glucose tolerance	sex	(I) BMI.30	(J) BMI.30	mean difference (I-J)	SE	Sig.[b]	95% Confidence Interval for the lower bound	upper bound
normoglycemia	female	BMI <30	BMI 30+	-4,446*	1.348	0.001	-7.095	-1.797
	male	BMI <30	BMI 30+	-8,138*	1.572	0.000	-11.227	-5.049
prediabetes	female	BMI <30	BMI 30+	-4.598	2.486	0.065	-9.484	0.289
	male	BMI <30	BMI 30+	-2.267	2.408	0.347	-6.999	2.465
type 2 diabetes	female	BMI <30	BMI 30+	-5,881*	1.551	0.000	-8.929	-2.832
	male	BMI <30	BMI 30+	-2.660	1.479	0.073	-5.567	0.246

Based on estimated marginal means
*. The mean difference is significant at level 0.05.
b.Adjustment for multiple comparisons: Bonferroni.

Pairwise comparisons

OUTCOME HCL

glucose tolerance	BMI.30	(I) sex	(J) sex	mean difference (I-J)	SE	Sig.[b]	95% Confidence Intervafor the lower bound	upper bound
normoglycemia	BMI <30	female	male	-0.808	0.759	0.287	-2.299	0.682
	BMI 30+	female	male	-4,501*	1.920	0.019	-8.273	-0.728
prediabetes	BMI <30	female	male	-1.836	2.362	0.437	-6.479	2.807
	BMI 30+	female	male	0.494	2.525	0.845	-4.468	5.457
type 2 diabetes	BMI <30	female	male	-1.692	1.550	0.276	-4.737	1.354
	BMI 30+	female	male	1.529	1.477	0.301	-1.374	4.432

Based on estimated marginal means
*. The mean difference is significant at level 0.05.
b.Adjustment for multiple comparisons: Bonferroni.

CARDIAC INDEX

Tests of between-subjects effects

OUTCOME Cardiac Index

Source	Type III Sum of Squares	df	Mean Square	F	Sig.
age	2.566	1	2.566	5.215	0.023
glucose tolerance	0.804	2	0.402	0.817	0.443
BMI.30	0.299	1	0.299	0.608	0.436
sex	0.106	1	0.106	0.216	0.643
glucose tolerance * BMI.30	1.359	2	0.679	1.381	0.253
glucose tolerance * sex	0.727	2	0.363	0.739	0.479
BMI.30 * sex	0.161	1	0.161	0.328	0.567
glucose tolerance * BMI.30 * sex	1.706	2	0.853	1.734	0.179

a. R Squared = 0,106 (Adjusted R Squared = 0,060)

Pairwise comparisons

OUTCOME Cardiac Index

BMI.30	sex	(I) glucose tolerance	(J) glucose tolerance	mean difference (I-J)	SE	Sig.[a]	95% Confidence Interval for the lower bound	upper bound
BMI <30	female	normoglycemia	prediabetes	-0.065	0.216	1.000	-0.586	0.457
			type 2 diabetes	0.279	0.204	0.518	-0.213	0.771
		prediabetes	normoglycemia	0.065	0.216	1.000	-0.457	0.586
			type 2 diabetes	0.344	0.249	0.506	-0.257	0.944
		type 2 diabetes	normoglycemia	-0.279	0.204	0.518	-0.771	0.213
			prediabetes	-0.344	0.249	0.506	-0.944	0.257
	male	normoglycemia	prediabetes	0.142	0.287	1.000	-0.549	0.833
			type 2 diabetes	0.249	0.217	0.754	-0.273	0.772
		prediabetes	normoglycemia	-0.142	0.287	1.000	-0.833	0.549
			type 2 diabetes	0.107	0.319	1.000	-0.663	0.877
		type 2 diabetes	normoglycemia	-0.249	0.217	0.754	-0.772	0.273
			prediabetes	-0.107	0.319	1.000	-0.877	0.663
BMI 30+	female	normoglycemia	prediabetes	0.171	0.326	1.000	-0.615	0.957
			type 2 diabetes	0.057	0.291	1.000	-0.644	0.759
		prediabetes	normoglycemia	-0.171	0.326	1.000	-0.957	0.615
			type 2 diabetes	-0.114	0.277	1.000	-0.781	0.554
		type 2 diabetes	normoglycemia	-0.057	0.291	1.000	-0.759	0.644
			prediabetes	0.114	0.277	1.000	-0.554	0.781
	male	normoglycemia	prediabetes	-0.746	0.384	0.160	-1.673	0.180
			type 2 diabetes	-0.339	0.328	0.909	-1.131	0.453
		prediabetes	normoglycemia	0.746	0.384	0.160	-0.180	1.673
			type 2 diabetes	0.407	0.308	0.562	-0.336	1.150
		type 2 diabetes	normoglycemia	0.339	0.328	0.909	-0.453	1.131
			prediabetes	-0.407	0.308	0.562	-1.150	0.336

Based on estimated marginal means
a.Adjustment for multiple comparisons: Bonferroni.

Pairwise comparisons

OUTCOME Cardiac Index

glucose tolerance	sex	(I) BMI.30	(J) BMI.30	mean difference (I-J)	SE	Sig.[a]	95% Confidence Interval for th lower bound	upper bound
normoglycemia	female	BMI <30	BMI 30+	0.151	0.254	0.552	-0.349	0.651
	male	BMI <30	BMI 30+	0.516	0.281	0.067	-0.037	1.069
prediabetes	female	BMI <30	BMI 30+	0.387	0.292	0.186	-0.188	0.961
	male	BMI <30	BMI 30+	-0.373	0.375	0.321	-1.111	0.366
type 2 diabetes	female	BMI <30	BMI 30+	-0.071	0.231	0.760	-0.525	0.384
	male	BMI <30	BMI 30+	-0.073	0.233	0.756	-0.531	0.386

Based on estimated marginal means
a.Adjustment for multiple comparisons: Bonferroni.

Pairwise comparisons

OUTCOME Cardiac Index

glucose tolerance	BMI.30	(I) sex	(J) sex	mean difference (I-J)	SE	Sig.[a]	95% Confidence Intervalfor the lower bound	upper bound
normoglycemia	BMI <30	female	male	0.060	0.130	0.643	-0.195	0.316
	BMI 30+	female	male	0.425	0.357	0.235	-0.278	1.129
prediabetes	BMI <30	female	male	0.267	0.325	0.411	-0.373	0.907
	BMI 30+	female	male	-0.492	0.346	0.156	-1.174	0.190
type 2 diabetes	BMI <30	female	male	0.031	0.238	0.897	-0.438	0.500
	BMI 30+	female	male	0.029	0.226	0.899	-0.416	0.474

Based on estimated marginal means
a.Adjustment for multiple comparisons: Bonferroni.

STROKE VOLUME

Tests of between-subjects effects

OUTCOME Stroke Volume

Source	Type III Sum of Squares	df	Mean Square	F	Sig.
age	760.139	1	760.139	7.206	0.008
glucose tolerance	441.791	2	220.895	2.094	0.126
BMI.30	74.912	1	74.912	0.710	0.400
sex	85.913	1	85.913	0.814	0.368
glucose tolerance * BMI.30	196.476	2	98.238	0.931	0.396
glucose tolerance * sex	23.160	2	11.580	0.110	0.896
BMI.30 * sex	32.474	1	32.474	0.308	0.580
glucose tolerance * BMI.30 * sex	131.105	2	65.552	0.621	0.538
Error	24,473.023	232	105.487		
Total	431,201.655	245			
Corrected Total	30,437.248	244			

a. R Squared = 0,196 (Adjusted R Squared = 0,154)

Pairwise comparisons

OUTCOME Stroke Volume

BMI.30	sex	(I) glucose tolerance	(J) glucose tolerance	mean difference (I-J)	SE	Sig.[a]	95% Confidence Interval for the lower bound	upper bound
BMI <30	female	normoglycemia	prediabetes	2.269	3.165	1.000	-5.364	9.903
			type 2 diabetes	6.392	2.986	0.100	-0.810	13.594
		prediabetes	normoglycemia	-2.269	3.165	1.000	-9.903	5.364
			type 2 diabetes	4.123	3.644	0.777	-4.666	12.911
		type 2 diabetes	normoglycemia	-6.392	2.986	0.100	-13.594	0.810
			prediabetes	-4.123	3.644	0.777	-12.911	4.666
	male	normoglycemia	prediabetes	5.268	4.196	0.632	-4.851	15.387
			type 2 diabetes	6.945	3.175	0.089	-0.713	14.602
		prediabetes	normoglycemia	-5.268	4.196	0.632	-15.387	4.851
			type 2 diabetes	1.676	4.674	1.000	-9.595	12.947
		type 2 diabetes	normoglycemia	-6.945	3.175	0.089	-14.602	0.713
			prediabetes	-1.676	4.674	1.000	-12.947	9.595
BMI 30+	female	normoglycemia	prediabetes	1.982	4.773	1.000	-9.528	13.492
			type 2 diabetes	1.590	4.262	1.000	-8.686	11.867
		prediabetes	normoglycemia	-1.982	4.773	1.000	-13.492	9.528
			type 2 diabetes	-0.392	4.051	1.000	-10.161	9.377
		type 2 diabetes	normoglycemia	-1.590	4.262	1.000	-11.867	8.686
			prediabetes	0.392	4.051	1.000	-9.377	10.161
	male	normoglycemia	prediabetes	-3.579	5.624	1.000	-17.142	9.984
			type 2 diabetes	2.435	4.809	1.000	-9.161	14.030
		prediabetes	normoglycemia	3.579	5.624	1.000	-9.984	17.142
			type 2 diabetes	6.014	4.512	0.552	-4.867	16.895
		type 2 diabetes	normoglycemia	-2.435	4.809	1.000	-14.030	9.161
			prediabetes	-6.014	4.512	0.552	-16.895	4.867

Based on estimated marginal means
a.Adjustment for multiple comparisons: Bonferroni.

Pairwise comparisons

OUTCOME Stroke Volume

glucose tolerance	sex	(I) BMI.30	(J) BMI.30	mean difference (I-J)	SE	Sig.[a]	95% Confidence Interval for the lower bound	upper bound
normoglycemia	female	BMI <30	BMI 30+	4.051	3.714	0.276	-3.266	11.369
	male	BMI <30	BMI 30+	4.934	4.111	0.231	-3.165	13.033
prediabetes	female	BMI <30	BMI 30+	3.764	4.269	0.379	-4.646	12.175
	male	BMI <30	BMI 30+	-3.914	5.490	0.477	-14.730	6.903
type 2 diabetes	female	BMI <30	BMI 30+	-0.750	3.378	0.825	-7.406	5.906
	male	BMI <30	BMI 30+	0.424	3.410	0.901	-6.294	7.142

Based on estimated marginal means
a.Adjustment for multiple comparisons: Bonferroni.

Pairwise comparisons

OUTCOME Stroke Volume

glucose tolerance	BMI.30	(I) sex	(J) sex	mean difference (I-J)	SE	Sig.[a]	95% Confidence Interval for the lower bound	upper bound
normoglycemia	BMI <30	female	male	-1.765	1.901	0.354	-5.510	1.980
	BMI 30+	female	male	-0.883	5.229	0.866	-11.184	9.419
prediabetes	BMI <30	female	male	1.234	4.756	0.796	-8.137	10.605
	BMI 30+	female	male	-6.444	5.067	0.205	-16.427	3.539
type 2 diabetes	BMI <30	female	male	-1.213	3.487	0.728	-8.083	5.657
	BMI 30+	female	male	-0.038	3.308	0.991	-6.555	6.479

Based on estimated marginal means
a.Adjustment for multiple comparisons: Bonferroni.

EJECTION FRACTION

Tests of between-subjects effects

OUTCOME EjectionFraction

Source	Type III Sum of Squares	df	Mean Square	F	Sig.
age	91.217	1	91.217	1.326	0.251
glucose tolerance	40.864	2	20.432	0.297	0.743
BMI.30	0.011	1	0.011	0.000	0.990
sex	44.840	1	44.840	0.652	0.420
glucose tolerance * BMI.30	31.978	2	15.989	0.232	0.793
glucose tolerance * sex	65.187	2	32.594	0.474	0.623
BMI.30 * sex	60.182	1	60.182	0.875	0.351
glucose tolerance * BMI.30 * sex	164.933	2	82.467	1.199	0.303

a. R Squared = 0,049 (Adjusted R Squared = 0,000)

Pairwise comparisons

OUTCOME Ejection Fraction

BMI.30	sex	(I) glucose tolerance	(J) glucose tolerance	mean difference (I-J)	SE	Sig.[a]	95% Confidence Interval for the lower bound	upper bound
BMI <30	female	normoglycemia	prediabetes	0.420	2.556	1.000	-5.744	6.584
			type 2 diabetes	2.808	2.411	0.737	-3.008	8.623
		prediabetes	normoglycemia	-0.420	2.556	1.000	-6.584	5.744
			type 2 diabetes	2.387	2.943	1.000	-4.709	9.484
		type 2 diabetes	normoglycemia	-2.808	2.411	0.737	-8.623	3.008
			prediabetes	-2.387	2.943	1.000	-9.484	4.709
	male	normoglycemia	prediabetes	1.230	3.388	1.000	-6.941	9.401
			type 2 diabetes	-3.113	2.564	0.678	-9.296	3.070
		prediabetes	normoglycemia	-1.230	3.388	1.000	-9.401	6.941
			type 2 diabetes	-4.344	3.774	0.753	-13.445	4.757
		type 2 diabetes	normoglycemia	3.113	2.564	0.678	-3.070	9.296
			prediabetes	4.344	3.774	0.753	-4.757	13.445
BMI 30+	female	normoglycemia	prediabetes	3.672	3.854	1.000	-5.622	12.966
			type 2 diabetes	1.756	3.441	1.000	-6.542	10.054
		prediabetes	normoglycemia	-3.672	3.854	1.000	-12.966	5.622
			type 2 diabetes	-1.916	3.271	1.000	-9.805	5.972
		type 2 diabetes	normoglycemia	-1.756	3.441	1.000	-10.054	6.542
			prediabetes	1.916	3.271	1.000	-5.972	9.805
	male	normoglycemia	prediabetes	0.628	4.542	1.000	-10.324	11.580
			type 2 diabetes	1.948	3.883	1.000	-7.415	11.312
		prediabetes	normoglycemia	-0.628	4.542	1.000	-11.580	10.324
			type 2 diabetes	1.320	3.644	1.000	-7.466	10.106
		type 2 diabetes	normoglycemia	-1.948	3.883	1.000	-11.312	7.415
			prediabetes	-1.320	3.644	1.000	-10.106	7.466

Based on estimated marginal means
a.Adjustment for multiple comparisons: Bonferroni.

Pairwise comparisons

OUTCOME Ejection Fraction

glucose tolerance	sex	(I) BMI.30	(J) BMI.30	mean difference (I-J)	SE	Sig.[a]	95% Confidence Interval for the lower bound	upper bound
normoglycemia	female	BMI <30	BMI 30+	0.559	2.999	0.852	-5.349	6.468
	male	BMI <30	BMI 30+	-2.745	3.319	0.409	-9.285	3.795
prediabetes	female	BMI <30	BMI 30+	3.811	3.447	0.270	-2.980	10.603
	male	BMI <30	BMI 30+	-3.347	4.433	0.451	-12.081	5.388
type 2 diabetes	female	BMI <30	BMI 30+	-0.492	2.728	0.857	-5.867	4.882
	male	BMI <30	BMI 30+	2.317	2.753	0.401	-3.108	7.742

Based on estimated marginal means
a.Adjustment for multiple comparisons: Bonferroni.

Pairwise comparisons

OUTCOME Ejection Fraction

glucose tolerance	BMI.30	(I) sex	(J) sex	mean difference (I-J)	SE	Sig.[b]	95% Confidence Interval for the lower bound	upper bound
normoglycemia	BMI <30	female	male	4,076*	1.535	0.008	1.052	7.100
	BMI 30+	female	male	0.772	4.222	0.855	-7.546	9.090
prediabetes	BMI <30	female	male	4.886	3.841	0.205	-2.681	12.453
	BMI 30+	female	male	-2.272	4.091	0.579	-10.333	5.789
type 2 diabetes	BMI <30	female	male	-1.845	2.816	0.513	-7.392	3.702
	BMI 30+	female	male	0.964	2.671	0.718	-4.298	6.227

Based on estimated marginal means
*. The mean difference is significant at level 0.05.
b.Adjustment for multiple comparisons: Bonferroni.

END OF DIASTOLE VOLUME

Tests of between-subjects effects

OUTCOME End of Diastole Volume

Source	Type III Sum of Squares	df	Mean Square	F	Sig.
age	3,470.265	1	3,470.265	13.009	0.000
glucose tolerance	1,441.086	2	720.543	2.701	0.069
BMI.30	331.195	1	331.195	1.242	0.266
sex	427.844	1	427.844	1.604	0.207
glucose tolerance * BMI.30	1,048.159	2	524.079	1.965	0.143
glucose tolerance * sex	478.731	2	239.365	0.897	0.409
BMI.30 * sex	46.799	1	46.799	0.175	0.676
glucose tolerance * BMI.30 * sex	301.633	2	150.817	0.565	0.569

a. R Squared = 0,297 (Adjusted R Squared = 0,260)

Pairwise comparisons

OUTCOME End of Diastole Volume

BMI.30	sex	(I) glucose tolerance	(J) glucose tolerance	mean difference (I-J)	SE	Sig.[b]	95% Confidence Interval for the lower bound	upper bound
BMI <30	female	normoglycemia	prediabetes	3.001	5.034	1.000	-9.137	15.140
			type 2 diabetes	7.633	4.749	0.328	-3.819	19.086
		prediabetes	normoglycemia	-3.001	5.034	1.000	-15.140	9.137
			type 2 diabetes	4.632	5.796	1.000	-9.344	18.608
		type 2 diabetes	normoglycemia	-7.633	4.749	0.328	-19.086	3.819
			prediabetes	-4.632	5.796	1.000	-18.608	9.344
	male	normoglycemia	prediabetes	9.569	6.673	0.459	-6.523	25.661
			type 2 diabetes	18,139*	5.050	0.001	5.962	30.315
		prediabetes	normoglycemia	-9.569	6.673	0.459	-25.661	6.523
			type 2 diabetes	8.570	7.432	0.750	-9.353	26.493
		type 2 diabetes	normoglycemia	-18,139*	5.050	0.001	-30.315	-5.962
			prediabetes	-8.570	7.432	0.750	-26.493	9.353
BMI 30+	female	normoglycemia	meandiffere	0.515	7.590	1.000	-17.788	18.819
			type 2 diabetes	0.519	6.777	1.000	-15.823	16.862
		prediabetes	normoglycemia	-0.515	7.590	1.000	-18.819	17.788
			type 2 diabetes	0.004	6.442	1.000	-15.531	15.539
		type 2 diabetes	normoglycemia	-0.519	6.777	1.000	-16.862	15.823
			prediabetes	-0.004	6.442	1.000	-15.539	15.531
	male	normoglycemia	prediabetes	-7.656	8.944	1.000	-29.224	13.913
			type 2 diabetes	3.202	7.647	1.000	-15.237	21.642
		prediabetes	normoglycemia	7.656	8.944	1.000	-13.913	29.224
			type 2 diabetes	10.858	7.176	0.395	-6.446	28.162
		type 2 diabetes	normoglycemia	-3.202	7.647	1.000	-21.642	15.237
			prediabetes	-10.858	7.176	0.395	-28.162	6.446

Based on estimated marginal means
*. The mean difference is significant at level 0.05.
b.Adjustment for multiple comparisons: Bonferroni.

Pairwise comparisons

OUTCOME End of Diastole Volume

glucose tolerance	sex	(I) BMI.30	(J) BMI.30	mean difference (I-J)	SE	Sig.[b]	95% Confidence Interval for the lower bound	upper bound
normoglycemia	female	BMI <30	BMI 30+	5.058	5.906	0.393	-6.578	16.693
	male	BMI <30	BMI 30+	14,827*	6.537	0.024	1.948	27.707
prediabetes	female	BMI <30	BMI 30+	2.572	6.788	0.705	-10.803	15.947
	male	BMI <30	BMI 30+	-2.397	8.730	0.784	-19.598	14.803
type 2 diabetes	female	BMI <30	BMI 30+	-2.056	5.372	0.702	-12.641	8.528
	male	BMI <30	BMI 30+	-0.109	5.422	0.984	-10.792	10.574

Based on estimated marginal means
*. The mean difference is significant at level 0.05.
b.Adjustment for multiple comparisons: Bonferroni.

Pairwise comparisons

OUTCOME End of Diastole Volume

glucose tolerance	BMI.30	(I) sex	(J) sex	mean difference (I-J)	SE	Sig.[b]	95% Confidence Interval for the lower bound	upper bound
normoglycemia	BMI <30	female	male	-10,204*	3.023	0.001	-16.159	-4.248
	BMI 30+	female	male	-0.434	8.315	0.958	-16.816	15.948
prediabetes	BMI <30	female	male	-3.636	7.564	0.631	-18.538	11.266
	BMI 30+	female	male	-8.605	8.057	0.287	-24.480	7.270
type 2 diabetes	BMI <30	female	male	0.302	5.545	0.957	-10.623	11.227
	BMI 30+	female	male	2.249	5.260	0.669	-8.114	12.613

Based on estimated marginal means
*. The mean difference is significant at level 0.05.
b.Adjustment for multiple comparisons: Bonferroni.

END OF SYSTOLE VOLUME

Tests of between-subjects effects

OUTCOME End of Systole Volume

Source	Type III Sum of Squares	df	Mean Square	F	Sig.
age	980.251	1	980.251	9.341	0.003
glucose tolerance	268.591	2	134.295	1.280	0.280
BMI.30	72.956	1	72.956	0.695	0.405
sex	130.259	1	130.259	1.241	0.266
glucose tolerance * BMI.30	349.202	2	174.601	1.664	0.192
glucose tolerance * sex	297.423	2	148.712	1.417	0.245
BMI.30 * sex	141.742	1	141.742	1.351	0.246
glucose tolerance * BMI.30 * sex	130.899	2	65.450	0.624	0.537

a. R Squared = 0,235 (Adjusted R Squared = 0,195)

Pairwise comparisons

OUTCOME End of Systole Volume

BMI.30	sex	(I) glucose tolerance	(J) glucose tolerance	mean difference (I-J)	SE	Sig.[b]	95% Confidence Interval for the lower bound	upper bound
BMI <30	female	normoglycemia	prediabetes	0.714	3.157	1.000	-6.899	8.328
			type 2 diabetes	1.227	2.979	1.000	-5.956	8.410
		prediabetes	normoglycemia	-0.714	3.157	1.000	-8.328	6.899
			type 2 diabetes	0.512	3.635	1.000	-8.253	9.278
		type 2 diabetes	normoglycemia	-1.227	2.979	1.000	-8.410	5.956
			prediabetes	-0.512	3.635	1.000	-9.278	8.253
	male	normoglycemia	prediabetes	3.974	4.185	1.000	-6.119	14.067
			type 2 diabetes	10,872*	3.167	0.002	3.235	18.510
		prediabetes	normoglycemia	-3.974	4.185	1.000	-14.067	6.119
			type 2 diabetes	6.898	4.662	0.421	-4.344	18.140
		type 2 diabetes	normoglycemia	-10,872*	3.167	0.002	-18.510	-3.235
			prediabetes	-6.898	4.662	0.421	-18.140	4.344
BMI 30+	female	normoglycemia	prediabetes	-1.465	4.761	1.000	-12.945	10.016
			type 2 diabetes	-1.236	4.251	1.000	-11.486	9.014
		prediabetes	normoglycemia	1.465	4.761	1.000	-10.016	12.945
			type 2 diabetes	0.228	4.041	1.000	-9.515	9.972
		type 2 diabetes	normoglycemia	1.236	4.251	1.000	-9.014	11.486
			prediabetes	-0.228	4.041	1.000	-9.972	9.515
	male	normoglycemia	prediabetes	-4.073	5.610	1.000	-17.601	9.455
			type 2 diabetes	0.296	4.796	1.000	-11.270	11.861
		prediabetes	normoglycemia	4.073	5.610	1.000	-9.455	17.601
			type 2 diabetes	4.369	4.501	0.998	-6.484	15.222
		type 2 diabetes	normoglycemia	-0.296	4.796	1.000	-11.861	11.270
			prediabetes	-4.369	4.501	0.998	-15.222	6.484

Based on estimated marginal means
*. The mean difference is significant at level 0.05.
b.Adjustment for multiple comparisons: Bonferroni.

Pairwise comparisons

OUTCOME End of Systole Volume

glucose tolerance	sex	(I) BMI.30	(J) BMI.30	mean difference (I-J)	SE	Sig.[b]	95% Confidence Interval for the lower bound	upper bound
normoglycemia	female	BMI <30	BMI 30+	0.990	3.704	0.790	-6.308	8.288
	male	BMI <30	BMI 30+	9,565*	4.100	0.021	1.487	17.643
prediabetes	female	BMI <30	BMI 30+	-1.189	4.258	0.780	-9.578	7.200
	male	BMI <30	BMI 30+	1.518	5.476	0.782	-9.271	12.306
type 2 diabetes	female	BMI <30	BMI 30+	-1.473	3.370	0.662	-8.112	5.166
	male	BMI <30	BMI 30+	-1.011	3.401	0.766	-7.712	5.689

Based on estimated marginal means
*. The mean difference is significant at level 0.05.
b.Adjustment for multiple comparisons: Bonferroni.

Pairwise comparisons

OUTCOME End of Systole Volume

glucose tolerance	BMI.30	(I) sex	(J) sex	mean difference (I-J)	SE	Sig.[b]	95% Confidence Interval for the lower bound	upper bound
normoglycemia	BMI <30	female	male	-8,128*	1.896	0.000	-11.864	-4.393
	BMI 30+	female	male	0.447	5.215	0.932	-9.828	10.721
prediabetes	BMI <30	female	male	-4.869	4.744	0.306	-14.215	4.478
	BMI 30+	female	male	-2.162	5.054	0.669	-12.119	7.795
type 2 diabetes	BMI <30	female	male	1.517	3.478	0.663	-5.335	8.369
	BMI 30+	female	male	1.979	3.299	0.549	-4.521	8.479

Based on estimated marginal means
*. The mean difference is significant at level 0.05.
b.Adjustment for multiple comparisons: Bonferroni.

MASS AVERAGE

Tests of between-subjects effects

OUTCOME MassAverage

Source	Type III Sum of Squares	df	Mean Square	F	Sig.
age	620.071	1	620.071	4.909	0.028
glucose tolerance	403.500	2	201.750	1.597	0.205
BMI.30	1,182.463	1	1,182.463	9.361	0.002
sex	2,830.405	1	2,830.405	22.406	0.000
glucose tolerance * BMI.30	388.771	2	194.386	1.539	0.217
glucose tolerance * sex	73.882	2	36.941	0.292	0.747
BMI.30 * sex	33.840	1	33.840	0.268	0.605
glucose tolerance * BMI.30 * sex	208.595	2	104.298	0.826	0.439
Error	29,307.127	232	126.324		
Total	963,466.829	245			
Corrected Total	41,029.289	244			

a. R Squared = 0,286 (Adjusted R Squared = 0,249)

Pairwise comparisons

OUTCOME MassAverage

BMI.30	sex	(I) glucose tolerance	(J) glucose tolerance	mean difference (I-J)	SE	Sig.[a]	95% Confidence Interval for the lower bound	upper bound
BMI <30	female	normoglycemia	prediabetes	1.311	3.464	1.000	-7.042	9.665
			type 2 diabetes	-4.345	3.268	0.555	-12.226	3.536
		prediabetes	normoglycemia	-1.311	3.464	1.000	-9.665	7.042
			type 2 diabetes	-5.657	3.988	0.472	-15.274	3.961
		type 2 diabetes	normoglycemia	4.345	3.268	0.555	-3.536	12.226
			prediabetes	5.657	3.988	0.472	-3.961	15.274
	male	normoglycemia	prediabetes	9.749	4.592	0.104	-1.324	20.823
			type 2 diabetes	-0.629	3.475	1.000	-9.008	7.751
		prediabetes	normoglycemia	-9.749	4.592	0.104	-20.823	1.324
			type 2 diabetes	-10.378	5.115	0.131	-22.712	1.956
		type 2 diabetes	normoglycemia	0.629	3.475	1.000	-7.751	9.008
			prediabetes	10.378	5.115	0.131	-1.956	22.712
BMI 30+	female	normoglycemia	prediabetes	-0.018	5.223	1.000	-12.613	12.578
			type 2 diabetes	-3.076	4.664	1.000	-14.322	8.170
		prediabetes	normoglycemia	0.018	5.223	1.000	-12.578	12.613
			type 2 diabetes	-3.059	4.433	1.000	-13.749	7.632
		type 2 diabetes	normoglycemia	3.076	4.664	1.000	-8.170	14.322
			prediabetes	3.059	4.433	1.000	-7.632	13.749
	male	normoglycemia	prediabetes	-3.316	6.155	1.000	-18.158	11.527
			type 2 diabetes	-0.831	5.262	1.000	-13.521	11.858
		prediabetes	normoglycemia	3.316	6.155	1.000	-11.527	18.158
			type 2 diabetes	2.484	4.938	1.000	-9.423	14.392
		type 2 diabetes	normoglycemia	0.831	5.262	1.000	-11.858	13.521
			prediabetes	-2.484	4.938	1.000	-14.392	9.423

Based on estimated marginal means
a.Adjustment for multiple comparisons: Bonferroni.

Pairwise comparisons

OUTCOME MassAverage

glucose tolerance	sex	(I) BMI.30	(J) BMI.30	mean difference (I-J)	SE	Sig.[b]	95% Confidence Interval for the lower bound	upper bound
normoglycemia	female	BMI <30	BMI 30+	-6.572	4.064	0.107	-14.579	1.435
	male	BMI <30	BMI 30+	-0.256	4.498	0.955	-9.119	8.606
prediabetes	female	BMI <30	BMI 30+	-7.901	4.671	0.092	-17.104	1.303
	male	BMI <30	BMI 30+	-13,321*	6.008	0.028	-25.158	-1.485
type 2 diabetes	female	BMI <30	BMI 30+	-5.303	3.697	0.153	-12.586	1.981
	male	BMI <30	BMI 30+	-0.459	3.731	0.902	-7.810	6.893

Based on estimated marginal means
*. The mean difference is significant at level 0.05.
b.Adjustment for multiple comparisons: Bonferroni.

Pairwise comparisons

OUTCOME MassAverage

glucose tolerance	BMI.30	(I) sex	(J) sex	mean difference (I-J)	SE	Sig.[b]	95% Confidence Interval for the lower bound	upper bound
normoglycemia	BMI <30	female	male	-13,722*	2.080	0.000	-17.820	-9.624
	BMI 30+	female	male	-7.407	5.722	0.197	-18.680	3.866
prediabetes	BMI <30	female	male	-5.284	5.205	0.311	-15.539	4.971
	BMI 30+	female	male	-10.705	5.545	0.055	-21.629	0.220
type 2 diabetes	BMI <30	female	male	-10,006*	3.816	0.009	-17.524	-2.488
	BMI 30+	female	male	-5.162	3.620	0.155	-12.293	1.970

Based on estimated marginal means
*. The mean difference is significant at level 0.05.
b.Adjustment for multiple comparisons: Bonferroni.
