# Supplementary material for: Sex differences in ectopic lipid deposits and cardiac function across a wide range of glycemic control: a secondary analysis
Source: Obesity (Silver Spring). 2024 Nov 18;32(12):2299–309. doi: 10.1002/oby.24153 (PMC11589534; doi:10.1002/oby.24153)
Supplement: Supplementary file 5 — Data S5. Supporting Information. [file OBY-32-2299-s004.pdf]

Supplementary Material 5

Full title: Sex differences in ectopic lipid deposits and cardiac function across a wide range of glycemic control: A secondary analysis

Authors: Jürgen Harreiter, PhD [1,2]*, Ivica Just, PhD [1,3]*, Michael Weber, PhD [4], Radka Klepochová, PhD[1,3], Magdalena Bastian, BSc [1], Yvonne Winhofer, PhD [1], Peter Wolf, PhD [1], Thomas Scherer, PhD [1], Michael Leutner, PhD [1], Lana Kosi-Trebotic, MD [1], Carola Deischinger, PhD [1], Marek Chmelík, PhD [3,5], Michael R Krebs, MD [1], Siegfried Trattnig, MD [3], Martin Krššák, PhD [1,3,#], Alexandra Kautzky-Willer, MD [1]

[1] Division of Endocrinology and Metabolism, Department of Internal Medicine III, Medical University of Vienna, Austria
[2] Department of Medicine, Landesklinikum Scheibbs, Austria
[3] High Field MR Center, Department of Biomedical Imaging and Image-guided Therapy, Medical University of Vienna, Austria
[4] Department of Biomedical Imaging and Image-guided Therapy, Medical University of Vienna, Austria
[5] Department of Technical Disciplines in Health Care at Faculty of Health Care, University of Prešov, Slovakia

* shared first authorship   # - correspondent author

Correspondence and reprint requests:
Martin Krššák, PhD,
Department of Internal Medicine III, Medical University in Vienna,
Währinger Gürtel 18-20, 1090 Vienna, Austria
E-Mail: martin.krssak@meduniwien.ac.at

Analysis of effect of age

In the main analysis, results are adjusted for age, using ANCOVA with age as a covariate. To show also the effect of age, we are providing further analysis.

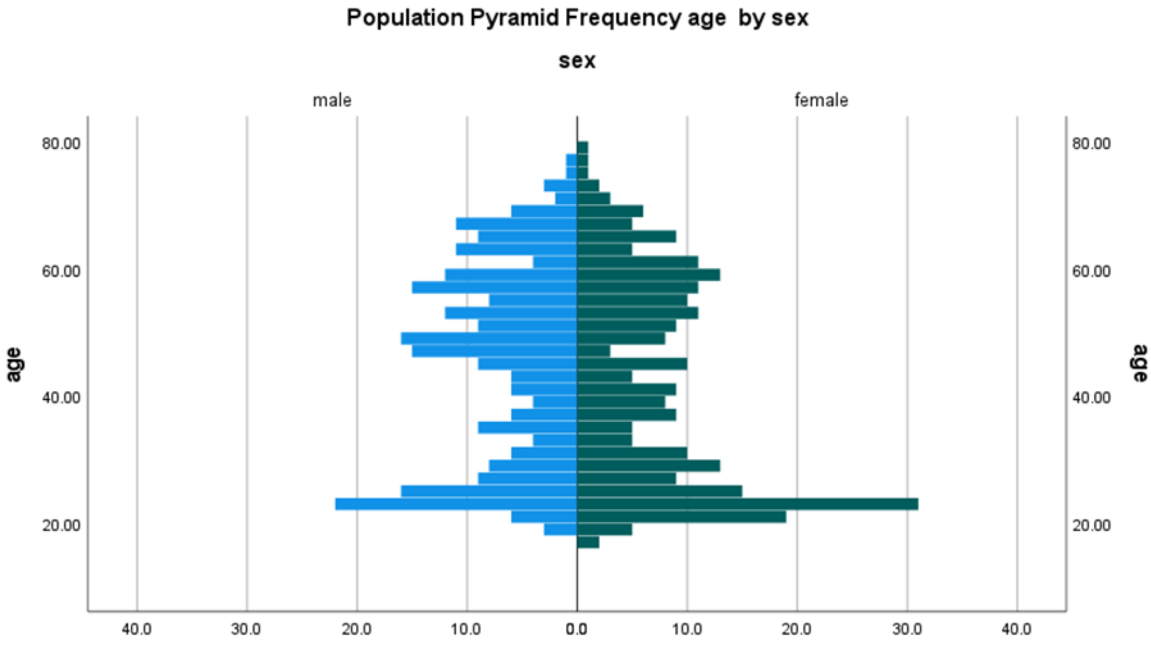

Fig.S5-1. Age distribution in both sexes

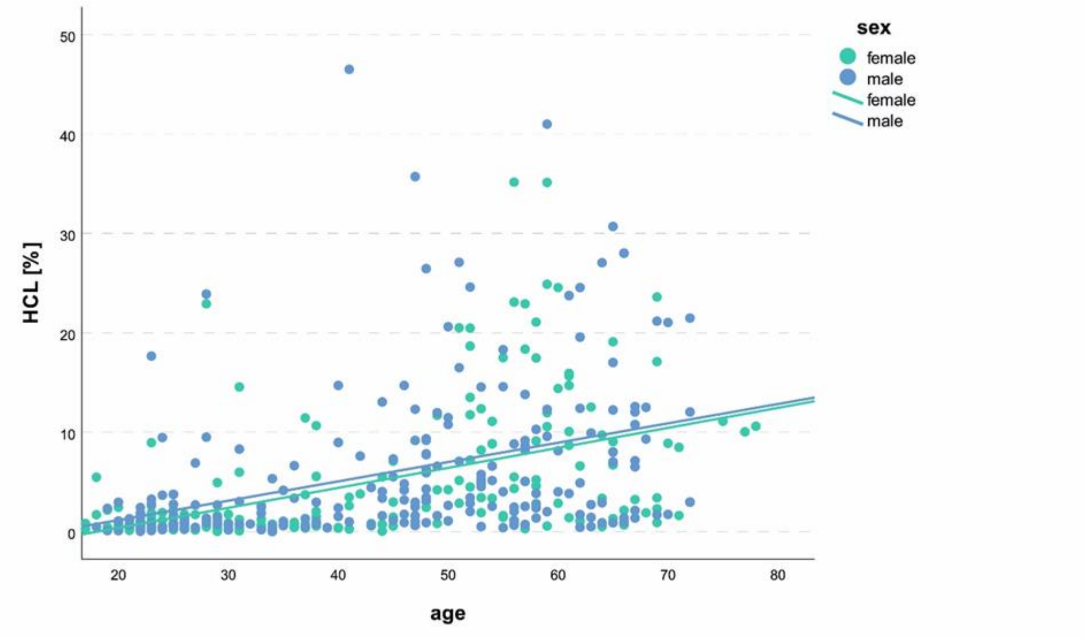

Fig.S5-2. Hepatocellular lipids (HCL) and age, in both men and women

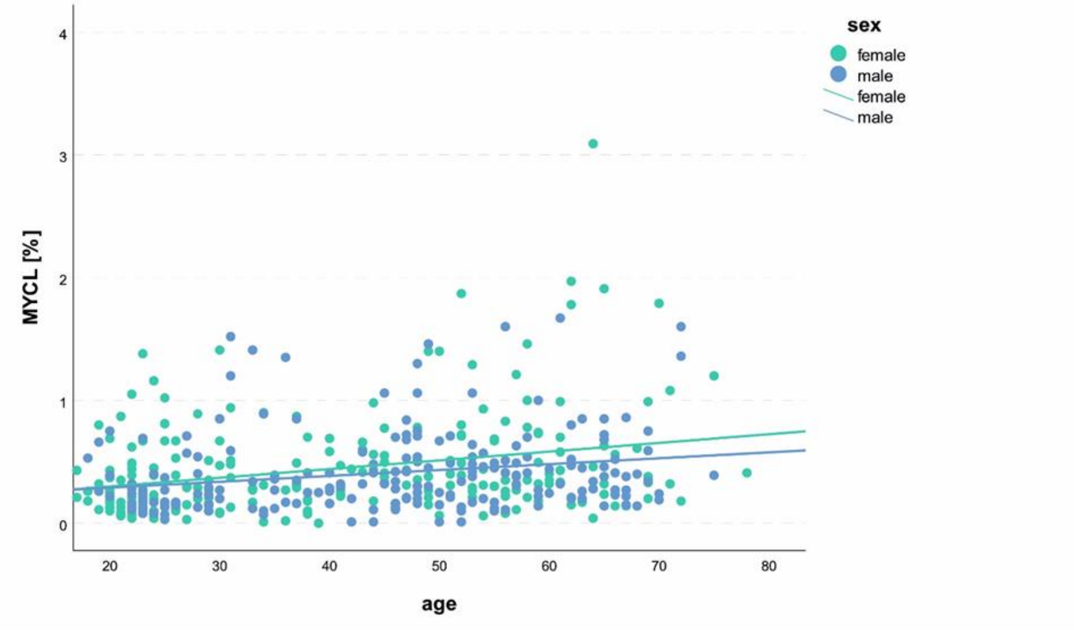

Fig.S5-3. Myocardial lipids (MYCL) and age, for both men and women

Correlation of HCL and MYCL with age is very similar for both sexes, with significance $p < 0.001$, but only weak to middle strength (Table S5-1).

Table S5-1. Correlations fo HCL and MYCL with age for men and women

		MYCL	HCL
Age female	Correlation coefficient	0,288**	0,604**
	Sig. (2-sided)	0.000	0.000
Age male	Correlation coefficient	0,283**	0,535**
	Sig. (2-sided)	0.000	0.000

We additionally evaluated age as predictor, its strength and its interaction with other parameters (BMI, glucose tolerance status and sex), assessing β-coefficients for all parameters including age. For MYCL, age is the strongest predictor (with the value of $\beta = 0.146$, Table S5-2) and is not moderated by any other parameter (Table S5-3).

Table S5-2. Beta coefficient for predictors of MYCL

Predictors of MYCL	β-coefficients
sex	-0,081
age	0,146
BMI	0,081
Glucose tolerance group1	0,095

Glucose tolerance group2	0,125

Table S5-3. Variables affecting MYCL with significances

Independent var. affecting MYCL	p-values
Glucose tolerance group	0,726
sex	0,570
BMI	0,267
age	0,013
Glucose tolerance group * age	0,430
sex * age	0,214
BMI * age	0,508

For HCL, age is not the strongest predictor (β=0.2050, as BMI is slightly stronger (β=0.302, Table S5-4). Effect of age is also moderated by both BMI and glucose tolerance status (Table S5-5).

Table S5-4. Predictors of HCL

Predictors of HCL	β-coefficients
Sex	0,059
Age	0,205
BMI	0,302
Glucose tolerance group1	0,026
Glucose tolerance group2	0,215

Table S5-5. Variables affecting HCL with significances

Independent var. affecting HCL	p-values
Glucose tolerance group	0,001
Sex	0,376
BMI.30	0,697
Age	0,286
Glucose tolerance group * age	0,018
sex * age	0,711
BMI.30 * age	0,051

For visual representation of glucose tolerance status groups, HCL and age correlations, see Fig S5-4.

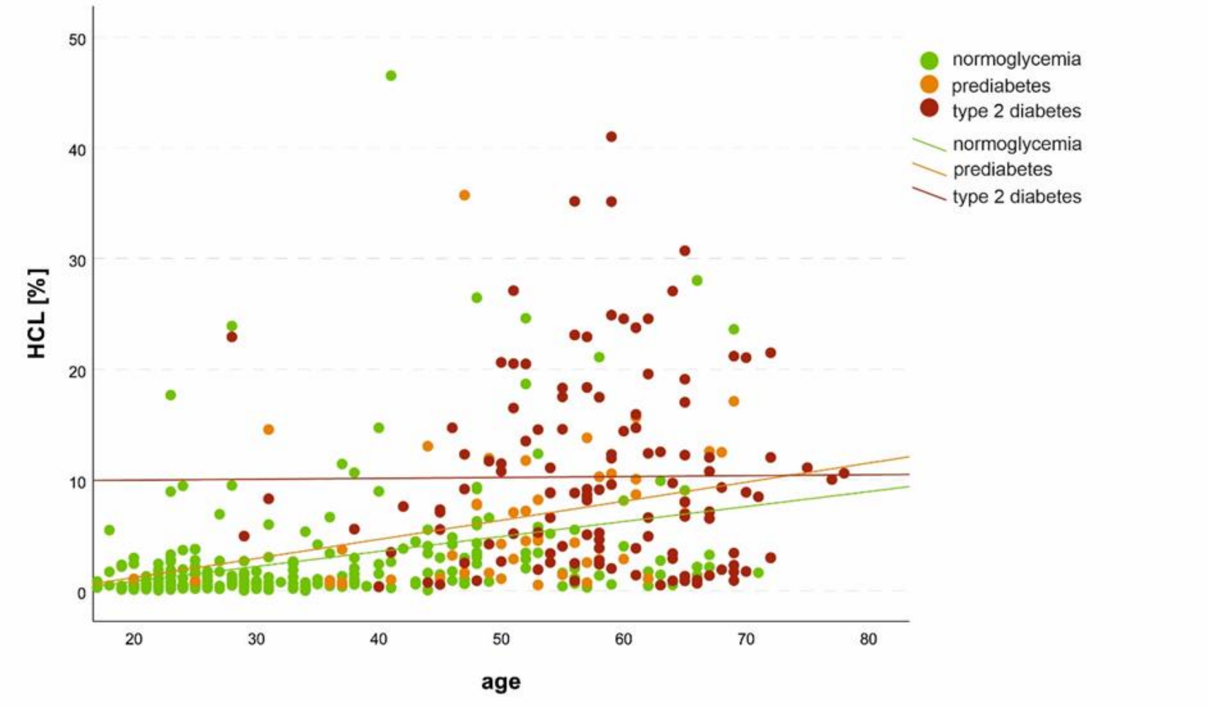

Fig. S5-4. Scatterplot of HCL and age for three groups based on glucose tolerance status

The strongest predictor for elevated HCL being BMI here is in accordance with the study results published by Williamson et al (Diabetes care 2011; 34(5):1139-1144) (1), where BMI was followed by the duration of diabetes and hbA1c in patients with type 2 diabetes. Also Lallukka et al (Sci Rep 7, 14561 (2017)) (2), showed that BMI, together with hbA1c is significant and independent predictor of liver fat in NAFLD patients.

References

1. Williamson RM, Price JF, Glancy S, Perry E, Nee LD, Hayes PC, et al. Prevalence of and risk factors for hepatic steatosis and nonalcoholic Fatty liver disease in people with type 2 diabetes: the Edinburgh Type 2 Diabetes Study. Diabetes Care. 2011;34(5):1139-44. Epub 2011/04/12. doi: 10.2337/dc10-2229. PubMed PMID: 21478462; PubMed Central PMCID: PMCPMC3114489.

2. Lallukka S, Sadevirta S, Kallio MT, Luukkonen PK, Zhou Y, Hakkarainen A, et al. Predictors of Liver Fat and Stiffness in Non-Alcoholic Fatty Liver Disease (NAFLD) - an 11-Year Prospective Study. Sci Rep. 2017;7(1):14561. Epub 2017/11/08. doi: 10.1038/s41598-017-14706-0. PubMed PMID: 29109528; PubMed Central PMCID: PMCPMC5674024.
